# Imbalance of pro- and anti-inflammatory cytokines in pulmonary sarcoidosis

**DOI:** 10.1155/S096293519600035X

**Published:** 1996-09

**Authors:** J. Müller-Quernheim

**Affiliations:** Medical Hospital Research Centre Borstel Parkallee 35 Borstel 23845 Germany


Mediators of Inflammation 5, 241–256 (1996)

### Imbalance of pro- and anti-inflammatory cytokines in pulmonary sarcoidosis

J. Müller-Quernheim

Medical Hospital, Research Centre Borstel, Parkallee 35, 23845 Borstel, Germany

### First Description and Search for Aetiology

A number of the characteristics of the disease known today as sarcoidosis were first described in the 18th and 19th centuries. In 1798 erythema nodosum was described by William, and as early as in 1882 the microscopical characteristics of epitheloid and giant cells were reported by Tenneson. In 1889 Ernest Besnier, a French dermatologist, presented a 34-year-old man with lesions on the face and upper limbs. He considered the facial changes related to lupus erythematodus, an autoimmune disorder characterized by facial skin lesions.[1] The term 'lupus pernio' shaped by Besnier is still used today to describe facial skin lesions of sarcoidosis patients.

Sarcoidosis is regarded today as a systemic disorder which can affect virtually all organs of the body. This view is based on the work of Kuznitzky, Bittorf and Schaumann. In 1914, Jörgen Schaumann, a Swedish dermatologist, wrote a prize-winning essay on lupus pernio, in which he expressed the view that Besnier's lupus pernio and Boeck's multiple sarcoids are manifestations of the same disease, that this disease might also involve the lymph nodes, the mucosa of the nose, the tonsils, the bones and the lungs, and that all of these manifestations are characterized by a histological pattern which he summarized as a 'tuberculoid granulomatous process'. Schaumann suggested the term 'lymphogranulomatosis benigna' for the disease since it appears to involve predominantly the lymphatic system. In this essay published in 1934 he identified radiographic changes of the bone due to sarcoidosis and typical histological changes in different organs, thus demonstrating that an affection of the skin is not mandatory for the diagnosis of sarcoidosis.[2,3]

As early as 1905 Boeck described sarcoidosis as 'a bacillary infectious disease, which is either identical with tuberculosis or closely related to it', however, proof of this hypothesis remains elusive. Nevertheless, there is an ongoing discussion of this question. Using methods of histology and molecular biology the presence of acid fast rods, mycobacterial DNA and mycobacterial rRNA has been demonstrated in sarcoidosis.[4–6] However, other investigators have not been able to reproduce these findings and, based on the assumption that per $10^6$ host cells of established lesions more than 15 organisms are required to play a pathogenetic role, it has been concluded that *Mycobacterium tuberculosis*, other mycobacteria, or closely related organisms are not involved in sarcoidosis,[7–10] recently reviewed by Mangiapan and Hance.[11]

In addition, a number of other infectious organisms have been claimed to be capable of inducing sarcoidosis. For example, mycoplasma-like organisms[12] or *Borrelia burgdorferi*[13] have recently been proposed as a possible cause of sarcoidosis, however, for the latter, data obtained by our group do not support this hypothesis.[14]

Epidemiological data and similarities with other infectious diseases support the hypothesis that sarcoidosis is induced by an infectious agent. Seasonal clustering of sarcoidosis in the months of June and July,[15] time and space clusters,[16,17] an increased incidence in health workers,[18] and the transmission of sarcoidosis by transplants[19] have been observed and further support the hypothesis of transmissible agents inducing sarcoidosis. Most interestingly, there are numerous reports of sarcoidosis patients who suffered from a relapse of sarcoidosis in a transplanted lung despite receiving immunosuppressive therapy.[20] *Vice versa* a patient receiving a lung from a donor who had a spontaneous remission of sarcoidosis in the past was observed to develop sarcoid-like lesions. These observations suggest

© 1996 Rapid Science Publishers that the aetiological agent hides within the lung and/or in other compartments of the body. Despite the efforts made to elucidate the aetiology the statement made by Longcope and Freimann in 1952 is still true: 'The aetiology of sarcoidosis is still obscure, the relation which it bears to tuberculosis or possibly other forms of granulomata remains a matter of contention and the conclusive demonstration of its presence rests entirely upon the histological structure of the lesions which it produces. ... The most telling contribution that could be made to our knowledge of this peculiar condition would be the discovery of its aetiology.'[21]

### Definition, Natural Course of the Disease, and Treatment

An evaluation of transbronchial or open lung biopsies of patients in the early stage of disease supports the clinical diagnosis by demonstrating the typical findings of noncaseating granulomas within the alveolar, bronchial and vascular walls.[22–24] These granulomas are diffusely scattered throughout the lung parenchyma. They are usually of varying age, ranging from highly cellular lesions to collections with diminishing cellularity, some fibrosis, and progressive hyalinization. Two characteristic zones can be seen in a typical, well-developed sarcoid granuloma: (i) a central zone or follicle which is tightly packed with cells, composed primarily of macrophages, multinucleated giant cells, and epitheloid cells; and (ii) a peripheral zone containing loosely arranged cells like a collar of lymphocytes, monocytes, and fibroblasts (Fig. 1).[22,25] Although many microscopic features may suggest sarcoidosis, the epitheloid granulomas, especially in their earlier stages, are indistinguishable from those of other idiopathic granulomatous disorders.

Thus, sarcoidosis is best defined in histopathological terms as 'a disease characterized by the presence in all of several affected organs and tissues of non-caseating epitheloid-cell granulomas, proceeding either to resolution or to conversion into hyaline connective tissue'.[22] The clinical diagnosis, however, can only be supported by typical histopathological findings. Pathognomonic criteria or a diagnostic 'golden standard' are absent.

Most patients with diagnosed sarcoidosis will undergo clinical and radiological resolution of the disease over a period ranging from several months to a few years.[26] A few of them develop a progressive form of the disease which may result in death.[27] Only rough estimates of the mortality of untreated sarcoidosis are available. If untreated a mortality of about 5% is estimated.

The aetiology of sarcoidosis remains unknown and, therefore, no specific treatment is possible. Fortunately, many patients will not require treatment because the symptoms are not disabling and frequently remit spontaneously. Prompt initiation of corticosteroid therapy, however, is indicated when granulomas cause dysfunction in vital organs or when the inflammation is particularly disabling or when disfiguring skin lesions emerge.[26]

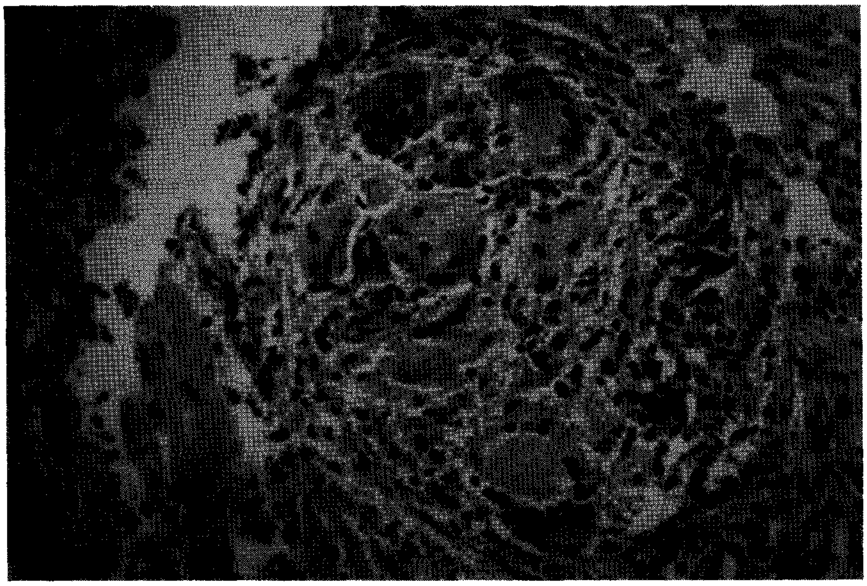

FIG. 1. Light photomicrograph of a typical non-caseating granuloma with multinucleated giant cells.[25]

### Bronchoalveolar Lavage

Although the technique of bronchoalveolar lavage (BAL) was introduced in the 1920s,[28] the first article describing pulmonary alveolar lavage as a method for harvesting large numbers of macrophages from the rabbit lung was not published until 1961 by Myrvik *et al.*[29] Finley *et al.*[30] have demonstrated that BAL can be safely performed in humans using a wedged catheter. With the introduction of the fibre-optic bronchoscope into clinical medicine by Ikeda *et al.*[31] BAL has become widely used for clinical investigations.[32,33] The observation of characteristic changes in the cytology of BAL in interstitial lung diseases first reported by Hunninghake in 1971 gave rise to a large number of detailed investigations of pulmonary immunology in health and disease.[34] The findings obtained by various researchers over the past 15 years form the basis of a concept of the immunopathogenesis of sarcoidosis discussed in the following.

### Ultrastructural Changes

In addition to granulomas, the morphological appearance of sarcoid of the lung has many of the features typical of mild interstitial lung diseases. Early in the disease unspecific ultrastructural lesions of the air–blood barrier can be observed, frequently even in areas of the lung which appear normal using light microscopy. The alveolar walls are expanded. The type I epithelial cells of the alveoli are injured denuding the basal membrane of a number of alveoli. These defects are covered by cuboidal cells, primarily pneumocytes type II. In other areas, the endothelial cells are damaged and capillaries are replaced by connective tissue. In rare cases a fibroblast proliferation in the alveolar and/or bronchial walls can be observed; it is these cases in which various amounts of dense fibrosis are observed replacing the normal parenchymal structures,[35] thus hindering the gas exchange.

Similar lesions can be observed in various clinical or experimental conditions including asbestosis,[36] idiopathic pulmonary fibrosis,[36,37] pulmonary histiocytosis X,[38] systemic sclerosis with lung involvement,[39] early stages of ARDS,[40] X-ray irradiation, paraquat, bleomycin, or 3-methylindole toxicity,[41,42] and exposure to nitrogen or oxygen.[41] The occurrence of similar reactions in such different conditions supports the concept of a common pattern of pulmonary response to diverse injuries.

A sequela of the early damage to the morphology of the basal membrane in interstitial lung diseases is a change in the composition of the alveolar lining fluid. Albumin and glucose are found at high concentrations in the alveolar lining fluid of patients with sarcoidosis[43] which may be due to leakage in the basal membrane or to a defect of the epithelial cells resulting in a decreased transmembrane transportation rate from the alveolar lumen back to the interstitium. These changes correlate with various parameters of inflammation, e.g. cellularity of BAL.[43] In addition, dramatic changes in the composition of the surfactant are observed in interstitial lung diseases, indicating an alteration in type II epithelial cells which produce the surfactant proteins and phospholipids.[44,45] These proteins are now recognized to exhibit immunomodulatory functions, either anti- or pro-inflammatory in nature.[44,46,47] The pathophysiological role of the interaction of epithelial lining fluid components and cells of the lower respiratory tract are unknown and represent a new and exciting field of research.

### Alveolar Macrophages

*Alveolar macrophage activation in sarcoidosis:* Due to a considerable increase in lavage cellularity, the absolute number of alveolar macrophages expands in sarcoidosis while the relative number decreases. The percentage of alveolar macrophages with monocytic appearance is elevated in sarcoidosis, suggesting a recent immigration of monocytic precursors of alveolar macrophages from the blood.[48–50] A number of cytokines chemotactic for monocytes, e.g. interleukin-1 (IL-1), tumour necrosis factor α (TNFα), macrophage inflammatory protein-1α (MIP-1α), and colony stimulating factors, are produced by alveolar cells in the course of inflammatory reactions of sarcoidosis and other interstitial lung diseases,[51–55] supporting the notion of monocyte immigration. However, those markers characteristic of a monocytic immunophenotype can be acquired by alveolar macrophages in the course of activation.[50] Thus, the question of monocyte immigration is not yet settled.

The activated state of these cells has been demonstrated on the basis of their spontaneous *in vitro* production of IL-1.[56] Results of the first report could not be reproduced by other investigators, although IL-1-mRNA was found in these cells.[57] A number of researchers have subsequently repeated these experiments and observed IL-1 in the supernatants of alveolar macrophages of patients with active sarcoidosis.[58–60] In addition, IL-1 inhibitors and IL-1 receptor antagonist were identified in BAL fluid and cell culture supernatants, thus providing at least a partial explanation of the conflicting results.[61,62]

Since the publication of the above results a number of monokines, i.e. TNFα, IL-6, MIP-1α, and monocyte chemotactic protein-1, have been identified as being released by alveolar macrophages in the course of sarcoidosis.[52,58–60,63,64] Analysing the release of TNFα and IL-6 by these cells revealed that the activation of the cells of the monocyte/macrophage lineage is compartmentalized, i.e. alveolar macrophages release these mediators spontaneously, whereas the corresponding cells of the peripheral blood are quiescent (Table 1).[60,63] In sarcoidosis TNFα is released at high concentrations at the place of the inflammatory reaction, however, a corresponding cachectin effect is absent in most patients, giving rise to the hypothesis that TNFα-binding or -neutralizing proteins or counteracting cytokines are simultaneously released. The fact that IL-6 proved in *in vitro* experiments to be capable of down-regulating the release of other pro-inflammatory cytokines, such as TNFα and IL-1,[65] led to the hypothesis that *in vivo* IL-6 might be able of dampening the alveolitis of sarcoidosis or even of inducing a spontaneous regression of the disease. However, the coexpression of TNFα and IL-6 in active sarcoidosis and the absence of IL-6 release of BAL cells from patients with inactive disease demonstrate (Table 1) that IL-6 does not exert detectable anti-inflammatory actions in sarcoidosis. Furthermore, soluble TNF-receptors (sTNF-R) may well be capable of counteracting TNFα effects.[66] Preliminary results obtained by our group demonstrate the presence of increased sTNF-R serum levels correlating with alveolar macrophage TNFα release and might, therefore, play a role in dampening the cachectin effects of TNFα (Fitschen, unpublished results). However, increased levels of both forms of the sTNF-R can be found in the alveolar lining fluid of patients with sarcoidosis and numerous interstitial and obstructive lung diseases[55] making it unlikely that they play a specific role in the immunopathogenesis of sarcoidosis.

Kinetic studies on the transcriptional level of the TNFα-gene revealed that maximal mRNA transcription is reached within 2 h after stimulation. Cytoplasmic TNFα was detected as early as 1 h after stimulation, culminating during the next 2 h to be followed by a decline.[67] Similar experiments with sarcoid alveolar macrophages revealed the transcription of the TNFα-gene at the time of BAL and its down-regulation during the next 24 h in cell culture.[60] Thus, the heightened spontaneous TNFα release seen in active sarcoidosis is the consequence of an *in vivo* activation step just prior to the removal of the cells from the lung indicating that the eliciting agent resides in the lower respiratory tract. In parallel to the findings on the transcriptional level the highest amount of TNFα is released during the first 3 h of *in vitro* culture followed by a sharp decline.[60] Therefore, it can be concluded that TNFα release is regulated in a physiological manner in those cells and that the putative agent causing sarcoidosis does not interfere with the regulation of the TNFα release neither on the transcriptional nor on the post-transcriptional level.

Subsequent cytokine studies were extended to

**Table 1.** Spontaneous release of TNFα and IL-6 by cells from patients with active or inactive pulmonary sarcoidosis*

| Cells | Spontaneous release of IL-6 (pg/ml/$10^6$ cells, median, range) | Spontaneous release of TNFα (pg/ml/$10^6$ cells, median, range) |
|---|---|---|
| Alveolar macrophages active sarcoidosis | 1012<br>104–3264<br>(*n* = 16) | 1156<br>100–17352<br>(*n* = 46) |
| Alveolar macrophages inactive sarcoidosis | 112<br>0–977<br>(*n* = 12) | 388<br>0–1643<br>(*n* = 27) |
| Alveolar macrophages controls** | 141<br>0–604<br>(*n* = 12) | 291<br>0–1011<br>(*n* = 26) |
| Peripheral blood mononuclear cells active sarcoidosis | 144<br>0–312<br>(*n* = 22) | 377<br>0–4122<br>(*n* = 41) |
| Peripheral blood mononuclear cells inactive sarcoidosis | 77<br>0–153<br>(*n* = 19) | 476<br>0–943<br>(*n* = 24) |
| Peripheral blood mononuclear cells controls | 91<br>0–124<br>(*n* = 16) | 306<br>0–887<br>(*n* = 23) |

* Inflammatory activity of disease was judged on the basis of clinical symptoms and organ dysfunction,[26] data from Refs 60 and 63, supplemented with unpublished data.

** Patients undergoing diagnostic bronchoscopy being retrospectively free of any inflammatory or malignant lung disease served as controls.

include the interstitium and the lymph node showing a simultaneous activation of alveolar macrophages, interstitial macrophages, and macrophages in the granulomas.[68] In general, interstitial macrophages differ from alveolar macrophages in the relative expression of immunological functions. The latter are poor accessory cells and IL-1 producers, but appear to be better equipped to clear infectious agents from the lower respiratory tract, whereas interstitial macrophages have higher accessory and immunoregulatory capabilities.[57,69–73] In marked contrast, the accessory capabilities of sarcoid alveolar macrophages have been found to be increased as measured in mixed lymphocyte reactions and antigen presentation.[73,74] However, the interpretation of these experiments has been complicated by possible stimulations via minor histocompatibility antigens or by intrinsic activities of autologous sarcoid T-cells present in the test. In a different cell culture system using a method insensitive to histo-incompatibilities, sarcoid alveolar macrophages were shown to express increased accessory functions mediated by adhesion molecules, e.g. CD80.[75]

*Down-regulation of alveolar macrophage activity:* The characteristics of the stimulated alveolar macrophages described above suggest an activation in the course of a normal immune response.[60] In approximately 60% of all sarcoid patients spontaneous regression of the disease is observed. Therefore, down-regulating mechanisms inducing spontaneous remission should be present in sarcoidosis.

Transforming growth factor-β (TGFβ) is a member of the superfamily of ubiquitous regulatory proteins which are necessary for cell growth, cell differentiation, and regulation of extracellular matrix production. Growing evidence also confirms its role as an immunomodulator, exhibiting pro-inflammatory and anti-inflammatory properties.[76–79] Interleukin-10 is another potent inhibitor of monocyte/macrophage- and T-cell-activation. It inhibits the cytokine production and proliferation of human monocytes and T cells[80–82] and the TNFα release of human pulmonary dendritic cells.[83] $CD4^+$ Th0 and $CD4^+$ Th2 cells are the major IL-10 producing cells[84,85] while macrophages and monocytes also release IL-10[80,86] although to a lesser extent.

IL-10 is capable of suppressing the release of TNFα and IL-1 by LPS activated alveolar macrophages in a dose-dependent manner. IL-6 release, however, is not affected by IL-10 (Fig. 2). TNFα and IL-1 are two key-mediators in sarcoid alveolitis required for the induction and maintenance of granulomata.[87] Thus, IL-10 is a candidate cytokine

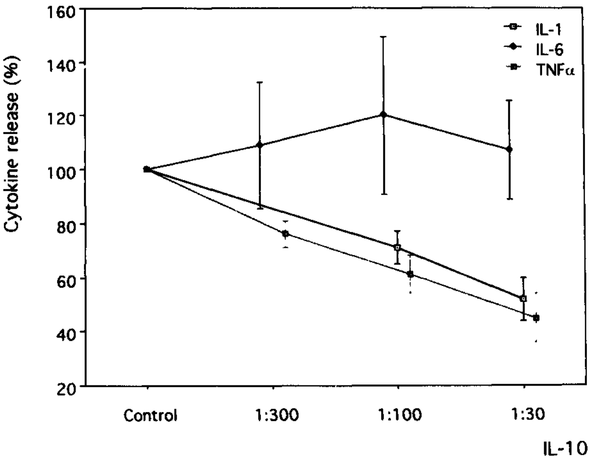

FIG. 2. Effects of IL-10 on cytokine release by stimulated alveolar macrophages ($n = 10$). Cells were stimulated with 1 μg LPS per ml in the presence of recombinant human (rh)IL-10. Controls were stimulated with LPS without rhIL-10. TNFα, IL-1α, and IL-6 concentrations were measured in the cell culture supernatants and expressed as a percentage of the LPS-stimulated control culture. The reductions of TNFα and IL-1α release are significant ($p < 0.001$ in all comparisons). IL-6 release was not influenced by the presence of IL-10 ($p > 0.2$). Taken from Zissel *et al.*[99]

for the induction of the spontaneous regression of sarcoidosis. Due to its TNFα suppressing capabilities high levels of IL-10 are anticipated in patients with low TNFα release and *vice versa*. As expected, in cell culture supernatants of alveolar macrophages of sarcoid patients containing high concentrations of TNFα counteracting IL-10 could not be found. In those supernatants with low concentrations of TNFα, however, in which an anti-inflammatory action has to be assumed IL-10 was absent indicating that the mechanism controlling the alveolitis does not employ this mediator.[88]

In an experimental protocol as described above for IL-10 a suppressive effect of TGFβ on LPS-activated, human alveolar macrophage cytokine release could not be detected. Employing lower concentrations of LPS, however, disclosed a dose-dependent suppressive effect of TGFβ on this type of cells. 100 ng/ml or 10 ng/ml of LPS are to potent stimulatory signals to be counteracted by TGFβ. Stimulation by 1 ng LPS/ml or *in vivo* stimulations resulting in an *in vitro* TNFα release result in an activation which can be down-regulated by TGFβ. By the presence of 200–1 000 pg TGFβ per ml the TNFα release of cells stimulated in such a way was significantly reduced to 65–89% of control cultures without TGFβ (Fig. 3).

In the lung the inactive form of TGFβ is an abundant cytokine[89,90] and mechanisms activating TGFβ can be observed during alveolitis. The release of proteases[91] and the acidification of the

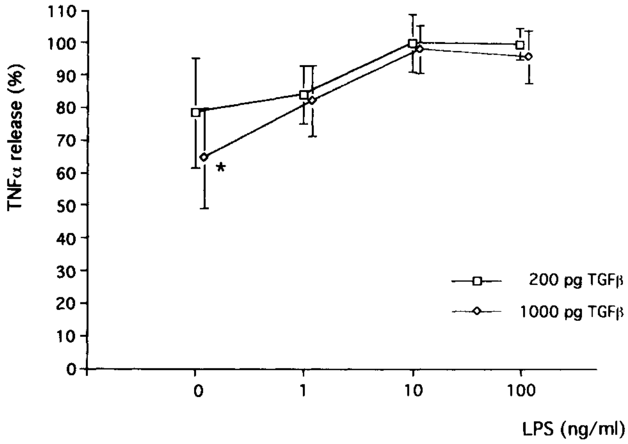

FIG. 3. Inhibition of TNFα release by alveolar macrophages ($n$ = 10) stimulated by various LPS doses. Cells either without *in vitro* stimulation or stimulated with 1, 10, or 100 ng LPS per ml were incubated with 200 or 1 000 pg TGFβ per ml. TNFα in cell culture supernatants was estimated by an ELISA and expressed as a percentage of control without TGFβ. The TNFα release suppressed by TGFβ at 0 ng LPS was stimulated *in vivo* by the disease of the respective patients (*$p < 0.05$). Taken from Zissel *et al.*[99]

micromilieu[92] by these cells are mechanisms known to activate TGFβ[90] and known to be present in sarcoid alveolitis.[71,90,93–96] In addition, cells of the monocytic type as can be found in sarcoid alveolitis[48,97] release preactivated TGFβ[98] and activated alveolar macrophages have been shown to down-regulate pro-inflammatory cytokine release in response to TGFβ.[99] This leads to the hypothesis that this cytokine might be involved in the down-regulation of sarcoid inflammation which was tested in a clinical study. For this purpose sarcoid patients were categorized into four groups: Group I consisted of patients with active disease exhibiting new or progressing symptoms. At the time of bronchoscopy indications for therapy were given and the patients received corticosteroids after bronchoscopy. Group II also exhibited signs of active disease at the time of clinical examination, however, due to the absence of indications no therapy was administered. After 6 months the supernatants of these twelve patients were allocated to Group II because spontaneous remission had occurred within the 6 months following the investigation. Patients with persisting signs of active disease, who did not receive therapy and showed no spontaneous remission were allocated to Group III. The patients in Group IV received either corticosteroid therapy or prednisolone plus azathioprine at the time of bronchoscopy.

Sarcoid patients whose BAL cells released low amounts of TGFβ either required corticosteroid therapy (Group I: 677 ± 159 pg/ml) or had evidence of persisting disease when no therapy was administered (Group III: 762 ± 419 pg/ml). In marked contrast, eight out of twelve patients with active disease who had a spontaneous remission of disease within the following six months (Group II: 1 422 ± 215 pg/ml) exhibited elevated TGFβ release by BAL cells. In these patients TGFβ release differed significantly from that recorded in Groups I, III and controls (II versus III: $p < 0.04$, II versus I and II versus control: $p < 0.004$). Most interestingly, in 5/5 patients under therapy elevated TGFβ release was also observed (Group IV, 1 698 ± 244 pg/ml) and differed significantly from Groups I, III and controls ($p < 0.04$ for all comparisons, Fig. 4).

Groups I (requiring therapy) and II (spontaneous remission) exhibited a markedly elevated TNFα release by BAL cells (1 952 ± 856 pg/ml and 2 374 ± 971 pg/ml, respectively) which proved to be significant in comparison with controls ($p < 0.02$ for both comparisons). The two other groups, persistent disease (Group III) and receiving therapy (Group IV) did not exhibit elevated TNFα release (326 ± 98 pg/ml and 123 ± 52 pg/ml, respectively, Fig. 4).

In addition, IL-2 concentrations were estimated in BAL cell culture supernatants of the four study groups. In Group I (requiring therapy) and in Group II (spontaneous remission) elevated IL-2 levels (9.8 ± 4.5 U/ml ($n$ = 12) and 2 ± 1.4 U/ml ($n$ = 5) respectively) without statistically significant differences were observed. In Group III (persistent disease) 3/3 patients had undetectable IL-2 levels. In Group IV (receiving corticosteroids), however, 1/3 patients showed an elevated IL-2 release (Fig. 4).

Although pro-inflammatory cytokines are considered to maintain sarcoid alveolitis[87] their presence is not associated with a state of disease requiring therapy if their action is counteracted by an anti-inflammatory mediator such as TGFβ, as can be seen from the difference in the course of the disease between Groups I and II in the above-described study.[88]

Considering the known immunomodulatory effects of TGFβ a direct anti-inflammatory action in sarcoid alveolitis seems conceivable. It can function as an autoregulatory lymphokine that limits lymphocyte expansion as it has been demonstrated for T-cells. After mitogen stimulation T-cells express the genes for pro-inflammatory cytokines as well as for TGFβ and accumulate TGFβ protein. However, its release is delayed consistent with a auto-regulatory role in limiting T-cell expansion[100,101] so that it can dampen the IL-1- and IL-2-responses of T-cells and therefore limit the inflammatory reac-

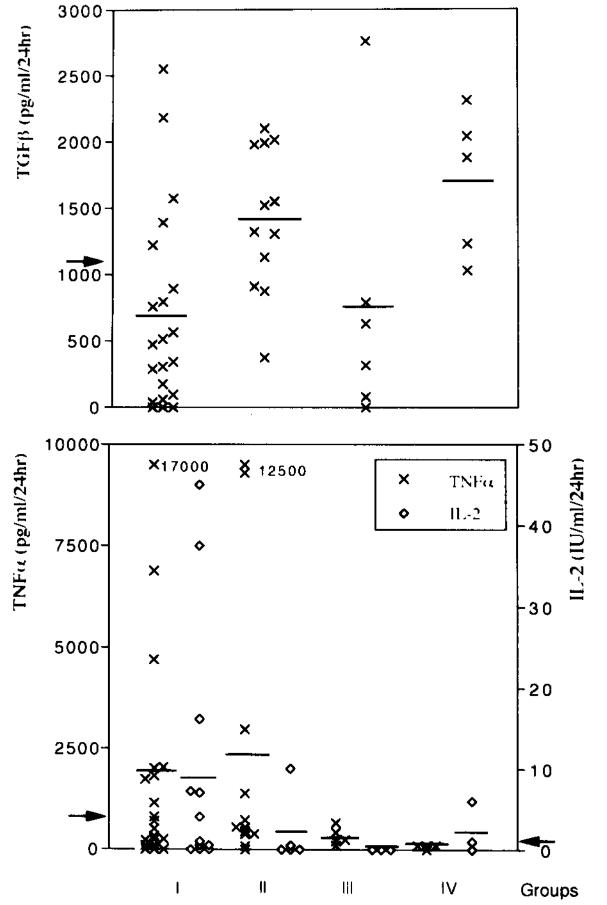

FIG. 4. Release of TGFβ (upper panel), TNFα and IL-2 (lower panel) by the four groups of sarcoid patients. Cytokines were measured in supernatants of cultured BAL cells ($10^6$ cells/ml, 24 h) either by bioassay (TGFβ and IL-2) or by ELISA (TNFα). Mean of TGFβ release is significantly elevated in Group II (spontaneous remission) and in Group IV (patients under therapy) indicated by bold lines. In Group I (requiring therapy) and Group III (persisting disease) TGFβ release is below the upper limit of the normal range. TNFα and IL-2 release is elevated in Group I and II whereas it is within the normal range in Groups III and IV. Upper limits of TGFβ, TNFα and IL-2 release derived from seven (TGFβ), 14 (TNFα) and 13 (IL-2) controls are indicated by the arrows (TNFα left, IL-2 right side). Taken from Zissel *et al.*[88]

tion.[76,102–104] This concept is further supported by findings in TGFβ knock-out mice. In these mice an excessive accumulation of inflammatory cells in several organs, including the lung, can be observed. In most animals death can be attributed to the effects of leukocyte infiltration in vital organs leading to the concept that TGFβ is required to counteract T-lymphocyte activation and expansion.[105,106] In pulmonary sarcoidosis this TGFβ effect seems to be involved in the induction of spontaneous regression of the disease.[88] Owing to the multiple TGFβ activating mechanisms in the micro-milieu of inflamed organs and the different status of receptor expression of the target cells it is extremely difficult to identify the role of TGFβ in a given immunopathological situation, discussed by Wahl.[107]

Macrophages can be deactivated by TGFβ and its mode of action is well described. In contrast to IL-10, which promotes degradation of pro-inflammatory cytokine mRNA, TGFβ inhibits post-transcriptional mechanisms in TNFα, IL-1α, and IL-1β production. It does not affect the level of TNFα mRNA, the release of preformed TNFα nor the degradation of TNFα. Thus, TGFβ appears to inhibit translation of TNFα mRNA.[7,91] In general, TGFβ enhances monocytic function which is mediated by TGF-receptors expressed in high density by these cells.[108] During the process of maturation to macrophages the receptor expression is down-regulated and the ligand binding to the remaining receptors induces deactivating mechanisms[108,109] making it feasible that the above described *in vitro* mechanisms act *in vivo*.

The patients in Group II of the study described above have a good prognosis and are certainly not under the risk of developing pulmonary fibrosis. High concentrations of TGFβ can, however, be observed in a number of *in vivo* and experimental situations resulting in fibrosis. Increased collagen synthesis in an animal model of hypersensitivity pneumonitis is associated with heightened TGFβ levels and can be abrogated by anti-TGFβ antiserum.[110] In bleomycin-treated mice, a model of pulmonary fibrosis, blocking of TGFβ by antibodies prevents fibrosis as well.[111,112] The foregoing data suggest that an increase in TGFβ concentration leads to fibrosis. Supporting this concept in chronic liver disease,[113,114] systemic sclerosis,[115] pulmonary fibrosis[114,116] and a number of other disorders with tissue fibrosis[117] such an increase of TGFβ has been identified. However, the response of fibroblasts to TGFβ differs with the state of disease and their expression of receptors for other cytokines like platelet-derived growth factor.[118] Fibroblasts of different organs differ in their response to TGFβ, e.g. lung fibroblasts down-regulate and scleroderma skin fibroblasts up-regulate their platelet-derived growth factor-receptors whereas normal skin fibroblasts do not modulate this receptor in response to TGFβ.[118] These findings are compatible with the concept that TGFβ, hence activated,[90] uses its anti-inflammatory capabilities demonstrated for macrophages,[99,119] T- and B-lymphocytes,[100,120,121] cell proliferation,[122] and cytokine release[123] to down-regulate pulmonary inflammation.

A study with inbred mice demonstrated that the susceptibility to the induction of pulmonary fibrosis by bleomycin sulphate is strain dependent. The lungs of bleomycin treated fibrosis susceptible C57BL/6J and fibrosis resistant BALB/cBy mice were analysed for their mRNA expression level of a panel of cytokines. TGFβ mRNA was found to increase seven-fold after bleomycin treatment in sensitive and three-fold in resistant animals. Analysis of F1 hybrids, which were shown in this report to be sensitive to bleomycin-induced fibrosis, revealed a segregation of a cytokine pattern completely concordant with the segregation of susceptibility phenotypes between the parental and F1 strains, i.e. the expression of IL-6 and TGFβ. This result indicates a possible association between sensitivity to bleomycin-induced fibrosis and inducibility of IL-6.[124] In this context it can be concluded that for the induction of fibrosis besides the presence of activated TGFβ a number of other immunobiological prerequisites, such as IL-6 expression, need to be fulfilled and it seems feasible that the association of TGFβ expression and good prognosis of sarcoid alveolitis is a crucial immunoregulatory phenomenon of this disorder. The notion of a potent anti-inflammatory action of TGFβ is further supported by the finding that highly immunogenic murine tumours transfected with TGFβ cDNA escape immunosurveillance.[125]

*Clinical parameters for the evaluation of macrophage activity:* Parameters suitable to gauge the activity of the cells of the macrophage/monocyte lineage in sarcoidosis have been delineated. Lysozym and angiotensin converting enzyme are products of epitheloid cells creating elevated serum levels in sarcoidosis. These serum levels reflect the granuloma burden of the entire body and can be used for a clinical assessment of the granulomatous inflammation.[126,127] These serum parameters are frequently used in everyday practice and their rise indicates an ongoing inflammation with an increasing number of granulomas.[126–128] The angiotensin converting enzyme concentration in alveolar lining fluid correlates with that of the serum and a number of parameters describing pulmonary inflammation, e.g. percentage of BAL lymphocytes.[129] However, this parameter is not specific and it can be found elevated in a number of interstitial lung diseases.[129,130]

CD14 is the most specific marker for cells of the monocyte/macrophage lineage[131–133] and its expression is age, race and gender independent.[134] Reports concerning expression of CD14 on alveolar macrophages in healthy volunteers and in sarcoidosis patients are controversial, however, its soluble form (sCD14) could serve as an ideal serum parameter to monitor the contribution of these cells to sarcoid inflammation. It has been shown in *in vitro* experiments that the expression of CD14 by pokeweed mitogen activated monocytes/macrophages is down-regulated by T-cells.[135] Furthermore, IL-4 and interferon-γ (IFNγ), both products of activated T-cells, have been confirmed to decrease CD14 expression on monocytes/macrophages by decreasing transcription of the CD14 gene.[133] It has been demonstrated that IFNγ is released spontaneously by T-lymphocytes of patients with active sarcoidosis[136] and that elevated levels of circulating IFNγ in serum can be detected in most untreated patients with sarcoidosis.[137]

In this context changes in CD14 expression by macrophages/monocytes and an increase in sCD14 may be expected in sarcoidosis patients. Sarcoid alveolar macrophages were found to express exaggerated levels of surface CD14[50,138] and in the alveolar lining fluid its soluble form is increased in active sarcoidosis.[139,140] Interestingly, a small increase in sCD14 serum levels correlating with neopterin and angiotensin converting enzyme was observed. Unfortunately, the changes in sCD14 serum concentration induced by sarcoid immunopathological mechanisms *in vivo* are of a magnitude which prevents the exploitation of this phenomenon for clinical purposes.[140]

Neopterin, a small, 250 Da metabolite of the guanosinetriphosphate pathway is released by activated macrophages and monocytes under the control of IFNγ produced by T-cells.[141] As expected, elevated serum levels were found in sarcoidosis and are used to monitor the activity of cells of the macrophage/monocyte lineage in the course of the disease.[140,142,143] Interestingly, a correlation between BAL cell TNFα or IL-6 release with serum neopterin could not be observed[63] giving rise to the hypothesis that the elevated neopterin levels are sequelae of cell activations in other body-compartments than the alveolar space, such as lymph nodes providing secreted molecules an easy access to the serum. Nevertheless, serum or urine neopterin concentration proved to be a very useful clinical parameter to probe the activity of the cells of the monocyte/macrophage lineage in the course of sarcoidosis.[140,142–144]

It has to be kept in mind that none of the above-mentioned parameters can be used to establish a diagnosis. Elevated levels are found in a number of diseases. Once the diagnosis of sarcoidosis has been made, the inflammatory activity of the immunopathogenetical processes described above can be monitored by the use of these serum parameters.

### T-cells

*T-cell activation:* Sarcoidosis is associated with an increase in the number of alveolar T-cells, and a shift to an increase in $CD4^+$ cells within these cells can be observed. In normal BAL up to 20% T-cells with a CD4/CD8 ratio ranging from 1.0 to 3.0 (median 2.2)[145] may be found. Apart from a small number of neutrophils (< 5%) the remaining cells are alveolar macrophages. In some cases of sarcoidosis more than 50% T-cells with a CD4/CD8 ratio > 10 can be observed exhibiting markers of activation, such as increased HLA-DR, VLA-1, and interleukin (IL)-2 receptor expression and capping of the T-cell-antigen receptor.[34,146–150]

Without any stimulation in tissue culture these T-cells have been found to release *in vitro* IL-2. This finding represents the first demonstration of the involvement of IL-2 in the immunopathophysiology of a human disease.[151,152] In spite of the systemic nature of the disease only the alveolar T-cells—but not those of the peripheral blood—secrete IL-2.[153] Interestingly, the regulation of the transcription of the IL-2 gene appears to be normal indicating a stimulation of the cells in a physiological fashion.[153] This view is supported by the finding of duBois *et al.* who demonstrated a capping of the T-cell-antigen receptor of alveolar T-cells in sarcoidosis suggesting a recent activation of the cells via this complex.[149] These two phenomena can be observed in cells of the BAL, indicating that the eliciting agent resides in the lung or that activated cells are attracted to the lung as observed in animal models of pulmonary inflammation.[154]

Owing to the fact that the genes of IL-2 and IL-2R are coexpressed the enumeration of IL-2R positive T-cells was considered to be an approach to estimate the number of activated alveolar T-cells. Only a moderate increase in $IL\text{-}2R^+$ T-cells was observed,[148,155,156] suggesting the presence of a small number of activated cells in the alveolar space or a dysregulation in the expression of the IL-2R. Results obtained by an *in vitro* study with sarcoid T-cells excluded the latter possibility.[148] Other immunological disorders, e.g. rheumatoid arthritis and multiple sclerosis, also exhibit small increases in $IL\text{-}2R^+$ T-cells at the site of inflammation, i.e. the synovial surface and the multiple sclerosis lesions, respectively, thus supporting the above-mentioned finding.[157,158] However, the milieu of the lower respiratory tract generated by pulmonary epithelial cells type II modulates the reactivity of the T-cells. In the presence of type II epithelial cells activated T-cells become arrested and do not progress in the cell cycle. When leaving this milieu the blockade is reversed, e.g. after migration of the T-cells to the lymph nodes.[159,160] The described scenario may also apply for sarcoidosis where a lymphocyte proliferation can be observed in sarcoid lymph nodes.[161]

The majority of the findings with regard to sarcoid T-cell response are characteristic of a T-cell mediated response to antigen and are highly suggestive of the presence of a persistent, poorly degradable antigen or antigens. In 1988 a bias towards an increased usage of the $V_\beta8$ region of the T-cell-antigen receptor in sarcoidosis peripheral blood and bronchoalveolar lavage T-lymphocytes was demonstrated, suggesting that T-cells accumulate secondary to external selective pressure, rather than in a random polyclonal fashion or by clonal expansion of one or a few T-cell clones.[162] This observation has been extended showing restricted usage of T-cell antigen receptor $V_\alpha$,[163] $V_\beta$,[164] and $C_\beta$-chains[165] in bronchoalveolar lavage and lung parenchyma.[166] However, other technical approaches have demonstrated an increased clonality in bronchoalveolar lavage cells without detecting preferred V-region families, leading to the assumption that the major source of T-cells is a polyclonal unspecific accumulation accompanied by a clonal expansion being different in every patient and contributing about 10% of the T-cells.[167–169] In normals an oligoclonality in the V-region usage of lung T-cell clones compared with blood clones could not be found.[170] In summary the sarcoidosis studies revealed a number $V_\alpha$- and $V_\beta$-family selections of the T-cell-antigen receptor in the detected clones, thus supporting the hypothesis of an unspecific stimulus. It can be concluded that the elusive agent or agents eliciting sarcoidosis select T-cell clones in the lung and other involved organs accompanied by an unspecific polyclonal T-cell accumulation as described for normal immune reactions.[171] This concept of compartmentalization is further supported by studies of autoimmune and chronic inflammatory diseases demonstrating a compartmentalized accumulation of T-cells with some restrictions in $V_\beta$-chain gene usage.[172–174]

The cytokine release by pulmonary lymphocytes is an immunopathological co-determinant of progressing alveolitis. Those patients with high numbers of activated, IL-2 releasing pulmonary T-cells have a high risk of developing pulmonary organ damage requiring corticosteroid therapy. Patients with low numbers of activated BAL lymphocytes or with quiescent cells differ significantly with lower percentages of progressing disease. Thus, parameters of immunopathogenesis can be used to gauge the inflammation of sarcoidosis and to judge prognosis. However, at present this approach requiring tissue culture is not applicable in everyday clinical practice.[175]

In a recent case report, the interesting course of a spontaneous improvement of bronchial asthma was described.[176] Bronchial asthma, a disease associated with activated T-helper cell(Th)-2-like lymphocytes releasing IL-4 and IL-5 in the lower respiratory tract in combination with a diminished or even missing IL-2 and IFNγ release[55,177,178] came to spontaneous improvement when the patient developed sarcoidosis a disorder characterized by activated Th1-like lymphocytes expressing Th1 cytokines like IL-2[151] and IFNγ[136] and the absence of Th2 cytokines like IL-4 and IL-5.[55] This observation supports the concept that similar to bronchial asthma an imbalance of Th1/Th2 cells might be the underlying immunopathogenetical mechanism which can be tested by identifying the cytokines released by T-cell clones derived from different body-compartments of patients with sarcoidosis.

In comparison with other body-compartments using lymphocytes from BAL or pulmonary parenchyma results in a low cloning efficiency.[179] Specific antigen,[180] which is not available for sarcoidosis, or unspecific stimulation[181] are required to obtain pulmonary T-cell clones. In comparison with blood the analysis of those clones obtained after unspecific stimulation of T-cells from sarcoid patients revealed that in pulmonary parenchyma a shift to Th1 cells can be observed. Moreover, clear-cut Th1, Th2, and Th0 cells were found but represented only a minority of the cells.[181–183] Most of the clones expressed the mRNA and secreted Th1 and Th2 cytokines simultaneously suggesting that they are of intermediate types as recently reviewed by Kelso.[184] Although only Th1 cytokines can be identified in BAL fluid[55] and BAL cell supernatants[151] of sarcoidosis patients their T-cells from pulmonary parenchyma or BAL are capable of releasing Th2 cytokines. Furthermore, T-cell clones from BAL disclosed in a rather high percentage clear-cut Th2 cells expressing only IL-4 and IL-5.[182] These findings support the hypothesis that in pulmonary sarcoidosis an imbalance of Th1/Th2 cytokines in favour of Th1 cytokines maintains the alveolitis. Th2 cells are present but rendered quiescent[55,182] and in case another stimulus activates these Th2 cells the sarcoid alveolitis might be down-regulated.

In this context it is of interest that an analysis on the single cell level of *in vitro* stimulated BAL T-cells from patients with allergic asthma revealed only a small percentage of cells belonging to the Th2 subset and the majority of cells were releasing IFNγ and IL-4 again demonstrating the frequent appearance of intermediate cytokine patterns in the human system.[185] Thus, the Th1/Th0/Th2 cells described in the murine system by Mosmann[186,187] contribute only a small percentage of the human T-cells and they represent polarised forms of the spectrum of T-cell cytokine expression used in the regulation of inflammatory reactions.[184,188]

In supernatants of BAL cells from sarcoid patients, cytokines preferentially produced by Th1 CD4$^+$ cells are frequently found[136,153] but not the Th2 cytokines IL-4 or IL-10.[55,88,183] This indicates that *in vivo* only active Th1 cells are present although Th2 can be elicited from this cell population *in vitro*.[182] In the literature considerations about the influence of co-stimulation on the development of Th1 and Th2 cells are controversial. There is some evidence that co-stimulation by CD80 decreases disease severity in experimental allergic encephalomyelitis by the activation of Th1 cells.[189,190] Additionally, it is reported that CD80/CD28 engagement increases the release of IL-2 more than the IL-4 release when the T-cell clones used are able to release both cytokines.[191] The preferential activation of Th1 cells observed in BAL of sarcoid patients may thus be a sequela of the exaggerated CD80 expression associated with an increased accessory function of alveolar macrophages in this disease.[75]

Another way of down-regulating T-cell activation is accomplished by anti-inflammatory cytokines like IL-10 and TGFβ and in the sarcoidosis study described above a negative correlation between TGFβ release by alveolar cells and their IL-2 release was noted ($r_s$ = –0.45, $p < 0.05$, Fig. 5).[88] Thus, the down-regulating capabilities of TGFβ on activated T-cells found in *in vitro* and animal studies[76,100–103] seem to take place *in vivo*. In this regard it is of interest that the activation of T-cells via CD28 is resistant to the down-regulation by TGFβ[103] explaining the manifestation of chronic disease resistant to immunosuppressive drugs in some patients. In a number of sarcoid patients an increased expression of CD80, the ligand of CD28, on alveolar macrophages has been demonstrated, indicating the

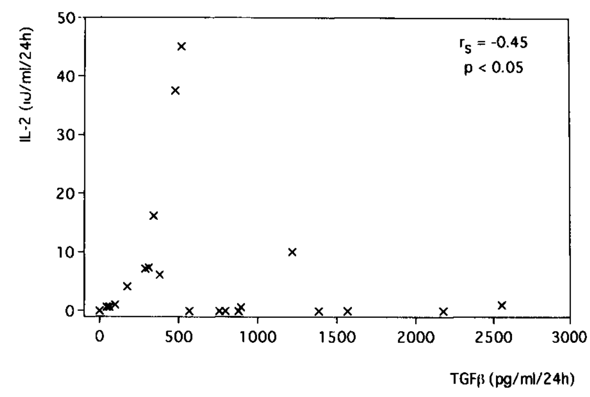

FIG. 5. Correlation of TGFβ and IL-2 release of alveolar immune cells in pulmonary sarcoidosis, taken from Zissel *et al.*[88]

possibility of an activation of alveolar T-cells via this pathway.[75,183] Thus, there is strong evidence that the course of the disease is determined by the mode of T-cell activation and the balance of pro- and anti-inflammatory cytokines in the micromilieu of the lower respiratory tract.

However, there are some findings which indicate that sarcoid T-cells exhibit altered characteristics of activation resulting in a heightened resistance to down-regulating mechanisms. Impaired G-proteins might inhibit adenyl hydrolysis leading to cAMP accumulation, thus maintaining the activated state of the cell.[192] In sarcoidosis and other lung diseases CD95 (Fas) is expressed by activated BAL T-cells.[193] Normal binding of the ligand CD70 (Fas-L) results in apoptosis.[194] Alterations of the Fas/Fas-L system, however, may result in the failure of T-cells to undergo apoptosis and an accumulation of activated T-cells at the sites of disease activity.[195] Other systems mediating apoptosis, as TNF-receptors with different intracytoplasmatic signal-transducing domains, have been identified and this type of receptors are expressed on the surface of sarcoid alveolar T-cells.[193,196] Thus, on the basis of the present data it cannot be concluded whether the T-cells become activated in the course of a normal immune response elicited by the unknown agent causing sarcoidosis or if the sarcoid T-cells induce the disease by failure of down-regulation after responding to a normal antigen.

*Clinical assessment of T-cell activation:* Activated T-cells express an IL-2R 55-kD/75-kD heterodimer on the cell surface and release a soluble form of the 55-kD chain (sIL-2R).[197,198] sIL-2R can be found in BAL fluid and serum of sarcoidosis patients and it is released by activated alveolar immune cells.[199–202] In addition to lymphocytes, macrophages are capable of expressing IL-2R upon activation[203] and it could be demonstrated that up to 50% of activated sarcoid alveolar macrophages exhibit increased numbers of IL-2R.[204] The relative contribution of lymphocytes and macrophages to the alveolar lining fluid sIL-2R concentration is not known.[201,204] A major contribution of these cells to the sIL-2R serum level cannot be expected because a leakage of the basal membrane or active transport mechanisms allowing a 55-kD protein to leave the alveolar space have not been observed in sarcoidosis.[35] In agreement with these findings the sIL-2R serum concentration was found to be independent of alveolar immune cell activation, indicating that these cells are not the main contributors to the exaggerated serum levels of sIL-2R.[201] Nevertheless, monitoring T-cell activity with sIL-2R

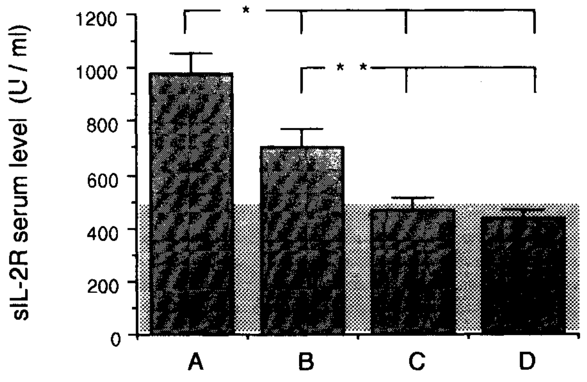

FIG. 6. Soluble IL-2 receptor serum concentration (mean ± SEM) at different stages of sarcoidosis. The sIL-2R serum levels were measured by a commercially available ELISA (DPC/Biermann, Bad Nauheim, Germany) and expressed as U/ml. According to clinical criteria the patients were categorized into four groups: A, active disease without therapy ($n$ = 39, 980 ± 74) ; B, active disease under corticosteroid medication (RX) ($n$ = 39, 708 ± 65); C, inactive disease without RX ($n$ = 38, 475 ± 44); D, inactive disease without RX ($n$ = 51, 434 ± 34). Statistically significant differences: * $p < 0.01$, ** $p < 0.05$; taken from Müller-Quernheim *et al.*[201] The normal range (mean ± 2SD) is indicated by the shaded area (In the mean time the manufacturer has changed the kit and the upper normal value is now 1 000 U/ml).

serum level reveals an intimate relationship between this parameter and the clinical activity of the disease, providing further evidence for the close linkage between the course of sarcoidosis and the activated state of T-cells (Fig. 6).[200–202,204,205] Moreover, those patients with high sIL-2R serum levels indicating a T-cell activation in the course of sarcoidosis exhibit a trend to a favourable spontaneous course of the disease with frequent spontaneous regressions and fewer indications for corticoid therapy.[206]

### Epithelial Cells

Epithelial cells of the lower respiratory tract, especially type II pneumocytes, are integrated in the pulmonary immune response as demonstrated by their constitutive expression of HLA-DR[207] and their expression of adhesion molecules such as CD54, CD51 and CD49d.[208,209] Furthermore, TNFα and IL-1, cytokines present in the lower respiratory tract in the course of sarcoidosis, are capable of inducing the release of monocyte chemoattractant protein-1 and IL-8 by pneumocytes II. In sarcoidosis the activation of these cells is indicated by their production of TGF-β,[210] their release of KL-6, a mucin-like protein glycoprotein,[211] and their up-regulation of HLA-DR expression.[212]

Although the involvement of pneumocytes II in the immunopathology of interstitial lung diseases has been demonstrated, their role in sarcoidosis remains to be elucidated. A number of methodological problems impede their investigation. The maintenance of pneumocytes II in tissue culture is difficult[213] and they can only be obtained from patients with interstitial lung diseases when a diagnostic open lung biopsy, currently a rare diagnostic procedure, is indicated. The available methods to isolate pneumocytes II require a large cell input to obtain sufficient numbers of pneumocytes II for cell biological experiments. For future research the preparation techniques need to be miniaturized to work with thoracoscopically obtained lung biopsies or even with transbronchial biopsies.

In respiratory physiology the most important product of pneumocytes II is the surface-active material of the alveolar lining fluid, called surfactant, which reduces surface tension at the alveolar surface, promotes lung expansion on inspiration and prevents lung collapse on expiration. It is composed of phospholipids and four apoproteins, named SP-A, B, C, and D.[214] Surfactant as a whole and especially the apoproteins have pro- and anti-inflammatory properties. In the presence of surfactant bacteria become opsonized,[215] macrophages are attracted by chemotaxis[47] and their phagocytosis is stimulated.[216] On the other hand surfactant protects the lower respiratory tract from harmful effects of the immune system by scavenging radical oxygen intermediates[217,218] and by inhibiting alveolar macrophage activation, e.g. the rise of intracellular calcium after activation is blocked by SP-A[44] and the activation of the genes of proinflammatory cytokines is inhibited on the transcriptional level.[219]

In interstitial lung diseases including sarcoidosis multiple alterations in the composition of surfactant are observed[220,221] resulting in a loss of macrophage inhibiting capabilities[44] or even stimulation of these cells.[220] Artificial surfactants with different amounts of apoproteins and phospholipids are now available as drugs can be delivered by inhalation.[222] A better understanding of the immunomodulatory capabilities of surfactant might open new therapeutical approaches for the treatment of chronic inflammation of the lower respiratory tract.

### Conclusion

Studies of BAL cells, T-cell clones, lymph nodes and lung parenchyma have advanced our understanding of the immunopathogenesis of interstitial lung diseases and, in particular, sarcoidosis. New clinical serum parameters allowing to monitor certain aspects of sarcoid immunopathogenesis have been delineated from this knowledge. Moreover, in the course of these investigations it became clear that the pulmonary immune reaction is compartmentalized. The activated cells, their mediators, and the corresponding control mechanisms are confined to the lung[60,63,136,149,153,156,161,169,193] although with sophisticated methods evidence for immune cell activation has been obtained from specimens of the peripheral blood.[137,223] Thus, organ-specific immunoregulatory mechanisms render the lung into a specialized immunological environment with a low responsiveness at the site of first antigen contact.[69,159] In spite of the fact that effective immune stimulation takes place in the lower respiratory tract only few of the activated cells undergo mitosis[151,153] but leave the lung[160,224] and proliferate in the regional lymph nodes.[161,225]

Studies of the pulmonary immune response in health and disease gave further support for this concept of compartmentalization. The immunoglobulin production of B-lymphocytes of the lung continues over a relatively long time after the lung is transplanted and seems to function independent of the immune system of the graft.[226] Immunophenotyping of the T-cells on the alveolar epithelial surface of healthy individuals revealed that these cells are permanently activated and exchange only very slowly with the blood pool[150,227] explaining the delayed changes of the diseased immunophenotypes when the patients are under therapy.[228] Local instillation of bacteria into the lung leads to neutrophil and cytokine accumulation in the area of instillation. If, however, the instillation dose is higher cells and mediators accumulate in the noninstilled parts of the lung as well and a systemic cytokine release is observed[229] indicating that the reactions to overwhelming injury cannot be confined to the lung as it can be seen from the systemic reaction, e.g. fever and myalgia, in sarcoidosis and other lung diseases.

### References

1. Besnier E. Lupus pernio de la face. *Ann Derm Syph* 1889; **10:** 333.
2. Schaumann J. Études sur le lupus pernio et ses raports avec les sarcoides et la tuberculose. *Ann Derm Syph* 1917; **5:** 357.
3. Schaumann J. 'Sur le lupus pernio', Mémoire présenté en Novembre 1914 à la Société française de Dermatologie et de Syphiligraphie pour le Prix Zambaco. Stockholm: 1934.
4. Vanek J, Schwarz J. Demonstration of acid-fast rods in sarcoidosis. *Am Rev Respir Dis* 1970; **101:** 395–400.
5. Saboor S, McI. Johnson N, McFadden J. Detection of mycobacterial DNA in sarcoidosis and tuberculosis with polymerase chain reaction. *Lancet* 1992; **339:** 1012–1015.
6. Mitchell I, Turk J, Mitchell D. Detection of mycobacterial rRNA in sarcoidosis with liquid-phase hybridisation. *Lancet* 1992; **339:** 1015–1017.
7. Bocart D, Lecossier D, de Lassence A, Valeyre D, Battesti J-P, Hance AJ. A search for mycobacterial DNA in granulomatous tissues from patients with sarcoidosis using the polymerase chain reaction. *Am Rev Respir Dis* 1992; **145:** 1142–1148.
8. Joyce-Brady M. Tastes great, less filling. *Am Rev Respir Dis* 1992; **145:** 986–987.

9. Gerdes J, Richter E, Rüsch-Gerdes S, *et al.* Mycobacterial nucleic acids in sarcoid lesions. *Lancet* 1992; **339:** 1536–1537.
10. Richter E, Greinert U, Kirsten D, *et al.* Assessment of mycobacterial DNA in cells and tissues of mycobacterial and sarcoid lesions. *Am J Respir Crit Care Med* 1996; **153:** 375–380.
11. Mangiapan G, Hance AJ. Mycobacteria and sarcoidosis: an overview and summary of recent molecular biology data. *Sarcoidosis* 1995; **12:** 20–37.
12. Putman CE, Baumgarten A, Gee JBL. The prevalence of mycoplasma complement-fixing antibodies in sarcoidosis. *Am Rev Respir Dis* 1975; **111:** 364–365.
13. Jacob F. Could *Borrelia burgdorferi* be a causal agent of sarcoidosis. *Medical Hypotheses* 1989; **30:** 241–243.
14. Martens H, Zöllner B, Schlaak M, Müller-Quernheim J. Keine erhöhte Prävalenz von Borrelia-brugdorferi-Antikörpern bei Sarkoidose. *Pneumologie* 1996; **50:** 77.
15. Badrinas F, Morera J, Fité E, Plasencia A. Seasonal clustering of sarcoidosis. *Lancet* 1989; **334:** 455–456.
16. Kern D, Neill M, Wrenn D, Varone J. Investigation of a unique time-space cluster of sarcoidosis in firefighters. *Am Rev Respir Dis* 1993; **148:** 974–980.
17. Parkes S, Baker SdC, Bourdillon R, Murray C, Rakshit M. Epidermiology of sarcoidosis in the Isle of Man -1: a case controlled study. *Thorax* 1987; **42:** 420–426.
18. Edmondstone W. Sarcoidosis in nurses: is there an association? *Thorax* 1988; **43:** 342–343.
19. Burke W, Keogh A, Maloney P, Delprado W, Bryant D, Spratt P. Transmission of sarcoidosis via cardiac transplantation. *Lancet* 1990; **336:** 1579.
20. Johnson BA, Duncan SR, Ohori NP, *et al.* Recurrence of sarcoidosis in pulmonary allograft recipients. *Am Rev Respir Dis* 1993; **148:** 1373.
21. Longcope W, Freiman O. A study of sarcoidosis. *Medicine* 1952; **31:** 132.
22. Mitchell D, Scadding J, Heard B, Hinson K. Sarcoidosis: histopathological definition and clinical diagnosis. *J Clin Path* 1977; **30:** 395–408.
23. Crystal RG, Bitterman PB, Rennard SI, Keogh BA. Interstial lung disease of unknown cause: disorders characterized by chronic inflammation of the lower respiratory tract. *N Engl J Med* 1984; **310:** 154–156, 235–244.
24. Crystal RG, Hunninhake GW, Gadek JE, Keogh BA, Rennard SI, Bitterman PB. The pathogenesis of sarcoidosis. In: Chretien J, Marsac J, Saltiel JC, eds. *Proc Int Conf Sarcoidosis and Other Granulomatous Disorders.* Paris: Peragamon Press, 1983: 13–35.
25. Cole S, Johnson K, Ward P. Pathology of sarcoidosis, granulomatous vasculitis, and other idiopathic granulomatous diseases of the lung. In: Fanburg B, ed. *Sarcoidosis and Other Granulomatous Diseases of the Lung.* Basel: Dekker, 1983: 149–202.
26. Hunninghake GW, Gilbert S, Pueringer R, *et al.* Outcome of the treatment of sarcoidosis. *Am J Respir Crit Care Med* 1994; **149:** 893–898.
27. Sones M, Israel H. Course and prognosis of sarcoidosis. *Am J Med* 1960; **29:** 64.
28. Jackson C. Bronchoscopy: past, present, and future. *N Engl J Med* 1928; **199:** 759–763.
29. Myrvik Q, Leake E, Farris B. Studies on pulmonary alveolar macrophages from the normal rabbit: a technique to procure them in a high state of purity. *J Immunol* 1961; **86:** 128–136.
30. Finley T, Swenson E, Curran W, Haber G, Ladman A. Bronchopulmonary lavage in normal subjects and patients with obstructive lung disease. *Ann Intern Med* 1967; **66:** 651–658.
31. Ikeda S, Yanai N, Ishikawa S. Flexible bronchofiberscope. *Keio J Med* 1968; **17:** 1–16.
32. Weinberger SE, Kelman JA, Elson NA, *et al.* Bronchoalveolar lavage in interstitial lung disease. *Ann Intern Med* 1978; **89:** 459–466.
33. Reynolds H, Newball H. Analysis of proteins and respiratory cells obtained from human lungs by bronchial lavage. *J Lab Clin Med* 1974; **84:** 559–573.
34. Hunninghake GW, Crystal RG. Pulmonary sarcoidosis: a disorder mediated by excess helper T-lymphocyte activity at sites of disease activity. *N Engl J Med* 1981; **305:** 429–434.
35. Planès C, Valeyre D, Loiseau A, Bernaudin J, Soler P. Ultrastructural alterations of the air-blood barrier in sarcoidosis and hypersensitivity pneumonitis and their relation to lung histopathology. *Am J Respir Crit Care Med* 1994; **150:** 1067–1074.
36. Corrin B, Dewar A, Rodriguez-Roisin R, Turner-Warwick M. Fine structural changes in cryptogenic fibrosing alveolitis and asbestosis. *J Pathol* 1985; **147:** 107–119.
37. Crystal R, Ferrans V, Basset F. Biologic basis of pulmonary fibrosis. In: Crystal R, West J, ed. *The Lung: Scientific Foundations.* New York: Raven Press, 1991: 2031–2046.
38. Fukuda Y, Basset F, Soler P, Ferrans V, Masugi Y, Crystal R. Intraluminal fibrosis and elastic fiber degradation lead to lung remodeling in pulmonary Langerhans cell granulomatosis (histiocytosis X). *Am J Pathol* 1990; **137:** 415–424.
39. Harrison NK, Myers AR, Corrin B, *et al.* Structural features of interstitial lung disease in systemic sclerosis. *Am Rev Respir Dis* 1991; **144:** 706–713.
40. Bachofen M, Bachofen H. Parenchymal changes. In: Crystal R, West J, ed. *The Lung: Scientific Foundations.* New York: Raven Press, 1991: 2019–2030.
41. Fine A, Goldstein R, Snider G. Animal models of pulmonary fibrosis. In: Crystal R, West J, ed. *The Lung: Scientific Foundations.* New York: Raven Press, 1991: 2047–2057.
42. Woods L, Wilson D, Schiedt M, Giri S. Structural and biochemical changes in lungs of 3-methylindole-treated rats. *Am J Pathol* 1993; **142:** 129–138.
43. Valeyre D, Soler P, Basset G, *et al.* Glucose, $K^+$, and albumin concentrations in the alveolar milieu of normal humans and pulmonary sarcoidosis patients. *Am Rev Respir Dis* 1991; **143:** 1096–1101.
44. Weissler J, Mendelson C, Moya F, Yarbrough W Jr. Effect of interstitial lung disease macrophages on T-cell signal transduction. *Am J Respir Crit Care Med* 1994; **149:** 191–196.
45. McCormack FX, King TEJ, Voelker DR, Robinson PC, Mason RJ. Idiopathic pulmonary fibrosis. Abnormalities in the bronchoalveolar lavage content of surfactant protein A. *Am Rev Respir Dis* 1991; **144:** 160–166.
46. Ansfield MJ, Benson BJ. Identification of the immunosuppressive components of canine pulmonary surface active material. *J Immunol* 1980; **122:** 125.
47. Hoffman RM, Claypool WD, Katyal SL, Singh G, Rogers RM, Dauber JH. Augmentation of rat alveolar macrophage migration by surfactant protein. *Am Rev Respir Dis* 1987; **135:** 1358–1362.
48. Hance AJ, Douches S, Winchester RJ, Ferrans VJ, Crystal RG. Characterization of mononuclear phagocyte subpopulations in the human lung by using monoclonal antibodies: changes in alveolar macrophage phenotype associated with pulmonary sarcoidosis. *J Immunol* 1985; **134:** 284–292.
49. Kiemle-Kallee J, Kreipe H, Radzun HJ, *et al.* Alveolar macrophages in idiopathic pulmonary fibrosis display a more monocyte-like immunophenotype and an increased release of free oxygen radicals. *Eur Respir J* 1991; **4:** 400–406.
50. Striz I, Wang J, Teschler H, Sorg C, Costabel U. Phenotypic markers of alveolar macrophage maturation in pulmonary sarcoidosis. *Lung* 1993; **171:** 293–303.
51. Hunninghake G, Glazier A, Monick M, Dinarello C. Interleukin-1 is a chemotactic factor for human T-lymphocytes. *Am Rev Respir Dis* 1987; **135:** 66–71.
52. Standiford T, Rolfe M, Kunkel S, *et al.* Macrophage inflammatory protein-1 α expression in interstitial lung disease. *J Immunol* 1993; **151:** 2852–2863.
53. Ming JW, Bersani L, Mantovani A. Tumor necrosis factor is chemotactic for monocytes and polymorphonuclear leukocytes. *J Immunol* 1987; **138:** 1469–1474.
54. Kreipe H, Radzun HJ, Heidorn K, *et al.* Proliferation, macrophage colony-stimulating factor, and macrophage colony-stimulating factor-receptor expression of alveolar macrophages in active sarcoidosis. *Lab Invest* 1990; **62:** 697–703.
55. Walker C, Bauer W, Braun R, *et al.* Activated T cells and cytokines in bronchoalveolar lavages from patients with various lung diseases associated with eosinophilia. *Am J Respir Crit Care Med* 1994; **150:** 1038–1048.
56. Hunninghake GW. Release of interleukin-1 by alveolar macrophages of patients with active pulmonary sarcoidosis. *Am Rev Respir Dis* 1984; **129:** 569–572.
57. Wewers MD, Saltini C, Sellers S, *et al.* Evaluation of alveolar macrophages in normals and individuals with active pulmonary sarcoidosis for the spontaneous expression of interleukin-1β gene. *Cell Immunol* 1987; **107:** 479–488.
58. Strausz J, Männel D, Pfeifer S, Borkowski A, Ferlinz R, Müller-Quernheim J. Spontaneous monokine release by alveolar macrophages in chronic sarcoidosis. *Int Archs Allergy Appl Immun* 1991; **96:** 68–75.
59. Steffen M, Petersen J, Oldigs M, *et al.* Increased secretion of tumor necrosis factor-alpha, interleukin-1-beta, and interleukin-6 by alveolar macrophages from patients with sarcoidosis. *Chest* 1993; **91:** 939–949.
60. Müller-Quernheim J, Pfeifer S, Männel D, Strausz J, Ferlinz R. Lung restricted activation of the alveolar macrophage/monocyte system in pulmonary sarcoidosis. *Am Rev Respir Dis* 1992; **145:** 187–192.
61. Nagai S, Aung H, Takeuchi M, Kusume K, Izumi T. IL-1 and IL-1 inhibitory activity in the culture supernatants of alveolar macrophages from patients with interstitial lung diseases. *Chest* 1991; **99:** 674–680.
62. Rolfe MW, Standiford TJ, Kunkel SL, *et al.* Interleukin-1 receptor antagonist expression in sarcoidosis. *Am Rev Respir Dis* 1993; **148:** 1378–1384.
63. Homolka J, Müller-Quernheim J. Increased interleukin 6 production by bronchoalveolar lavage cells in patients with active sarcoidosis. *Lung* 1993; **171:** 173–183.
64. Baughman RP, Strohofer SA, Buchsbaum J, Lower EE. Release of tumor necrosis factor by alveolar macrophages of patients with sarcoidosis. *J Lab Clin Med* 1990; **115:** 36–42.
65. Zitnik R, Elias JA. Interleukin-6 and the lung. In: Kelley J, ed. *Cytokines of the Lung.* New York: Marcel Dekker, 1993: 229–280.
66. Seckinger P, Zhang JH, Hauptmann B, Dayer JM. Characterisation of tumor necrosis factor α (TNF-α) inhibitor: evidence of immunological cross-reactivity with the TNF receptor. *Proc Natl Acad Sci USA* 1990; **87:** 5188–5192.

67. Rosenblum GR, Donato NJ. Tumor necrosis factor α: a multifaceted hormone. *Crit Rev Immunol* 1989; **9:** 21–44.
68. Devergne O, Emilie D, Peuchmaur M, Crevon MC, D'Agay MF, Galanaud P. Production of cytokines in sarcoid lymph nodes: preferential expression of interleukin-1β and interferon-γ genes. *Hum Pathol* 1992; **23:** 317–323.
69. Lohmann-Matthes M, Steinmüller C, Franke-Ullmann G. Pulmonary macrophages. *Eur Respir J* 1994; **7:** 1678–1689.
70. Nicod LP, Lipscomb MF, Weissler JC, Lyons CR, Alberton J, Toews GB. Mononuclear cells in human lung parenchyma. Characterization of a potent accessory cell not obtained by bronchoalveolar lavage. *Am Rev Respir Dis* 1987; **136:** 818–823.
71. Fels A, Cohn Z. The alveolar macrophage. *J Appl Physiol* 1986; **60:** 353–369.
72. Toews GB, Lem VM, Weissler JC, *et al.* Antigen presentation by alveolar macrophages in patients with sarcoidosis. *Ann NY Acad Science* 1986; **465:** 74–81.
73. Lem VM, Lipscomb MF, Weissler JC, *et al.* Bronchoalveolar cells from sarcoid patients demonstrate enhanced antigen presentation. *J Immunol* 1985; **135:** 1766–1771.
74. Venet A, Hance AJ, Saltini C, Robinson BWS, Crystal RG. Enhanced alveolar macrophage-mediated antigen-induced T lymphocyte proliferation in sarcoidosis. *J Clin Invest* 1985; **75:** 293–301.
75. Müller-Quernheim J, Zissel G. Elevated accessory function of alveolar macrophages in pulmonary sarcoidosis. *Am J Respir Crit Care Med* 1994; **149:** A262.
76. Wahl SM, Hunt DA, Wong HL, *et al.* Transforming growth factor-β is a potent immunosuppressive agent that inhibits IL-1-dependent lymphocyte proliferation. *J Immunol* 1988; **140:** 3026–3032.
77. Wahl SM, McCartney-Francis N, Mergenhagen S. Inflammatory and immunoregulatory roles of TGF-β. *Immunol Today* 1989; **10:** 258–261.
78. Bull HG, Thornberry NA, E.H. C. Purification of angiotensin-converting enzyme from rabbit lung and human plasma by affinity chromatography. *J Biol Chem* 1985; **260:** 2963–2972.
79. Ehlers MRW, Maeder DL, Kirsch RE. Rapid affinity chromatographic purification of human lung and kidney angiotensin-converting enzyme with the novel N-carboxyalkyl dipeptide inhibitor N- (1-carboxy-5-aminopentyl) glycylglycine. *Biochem Biophys Acta* 1986; **883:** 361.
80. de Waal Malefyt R, Haanen JBAG, Spits H, *et al.* Interleukin 10 (IL-10) and viral IL-10 strongly reduce antigen-specific human T cell proliferation by diminishing the antigen-presenting capacity of monocytes via downregulation of class II major histocompatibility complex expression. *J Exp Med* 1991; **174:** 915–924.
81. de Waal Malefyt R, Yssel H, de Vries JE. Direct effects of Il-10 on subsets of human $CD4^+$ T cell clones and on resting T cells. *J Immunol* 1993; **150:** 4754–4765.
82. Taga K, Tosato G. Il-10 inhibits human T cell proliferation and Il-2 production. *J Immunol* 1992; **148:** 1143–1148.
83. Nicod L, El Habre F, Dayer J, Boehringer N. Interleukin-10 decreases tumor necrosis factor α and β in alloreactions induced by human dendritic cells and macrophages. *Am J Respir Cell Mol Biol* 1995; **13:** 83–90.
84. Yssel H, de Waal Malefyt R, Roncarolo M-G, *et al.* Il-10 is produced by subsets of human $CD4^+$ T cell clones and peripheral blood T cells. *J Immunol* 1992; **149:** 2378–2384.
85. Street NE, Mosmann TR. Functional diversity of T lymphocytes due to secretion of different cytokine patterns. *FASEB Journal* 1991; **5:** 171–177.
86. Fiorentino DF, Zlotnik A, Mosmann TR, Howard M, O'Garra A. IL-10 inhibits cytokine production by activated macrophages. *J Immunol* 1991; **147:** 3815–3822.
87. Kunkel SL, Chensue SW, Strieter RM, Lynch JP, Remick DG. Cellular and molecular aspects of granulomatous inflammation. *Am J Respir Cell Mol Biol* 1989; **1:** 439–447.
88. Zissel G, Homolka J, Schlaak J, Schlaak M, Müller-Quernheim J. Antiinflammatory cytokine release by alveolar macrophages in pulmonary sarcoidosis. *Am J Respir Crit Care Med* 1996; **154**.
89. Yamauchi K, Martinet Y, Basset P, Fells GA, Crystal RG. High levels of transforming growth factor-β are present in the epithelial lining fluid of the normal human lower respiratory tract. *Am Rev Respir Dis* 1988; **137:** 1360–1363.
90. Kelley J. Transforming growth factor-β. In: Kelley J, ed. *Cytokines.* New York: Marcel Dekker, 1993: 101–127.
91. Tsunawaki S, Sporn M, Ding A, Nathan C. Deactivation of macrophages by transforming growth factor-β. *Nature* 1988; **334(21):** 260–262.
92. Silver IA, Murrills RJ, Etherington DJ. Microelectrode studies on the acid microenvironment beneath adherent macrophages and osteoclasts. *Exp Cell Res* 1988; **175:** 266–276.
93. Fels AOS, Nathan CF, Cohn ZA. Hydrogen peroxide release by alveolar macrophage from sarcoid patients and by alveolar macrophage from normals after exposure to recombinant interferon α, β, and γ and 1,25-dihydroxyvitamin D3. *J Clin Invest* 1987; **80:** 381–386.
94. Schaberg T, Rau M, Stephan H, Lode H. Increased number of alveolar macrophages expressing surface molecules of the CD11/CD18 family in sarcoidosis and idiopathic fibrosis is related to the production of superoxide anions by these cells. *Am Rev Respir Dis* 1993; **147:** 1507–1513.
95. Dalhoff K, Bohnet S, Braun J, Kreft B, Wießmann K. Intercellular adhesion molecule 1 (ICAM-1) in the pathogenesis of mononuclear cell alveolitis in pulmonary sarcoidosis. *Thorax* 1993; **48:** 1140–1144.
96. Aerts C, Wallaert JM, Grosbois JM, Voisin C. Release of superoxide anion by alveolar macrophages in pulmonary sarcoidosis. *Ann NY Acad Science* 1986; **465:** 193–200.
97. Hamacher J, Schaberg T. Adhesion molecules in lung diseases. *Lung* 1994; **172:** 189–213.
98. Grotendorst GR, Smale G, Pencev D. Production of transforming growth factor-β. *J Cell Physiol* 1989; **140:** 396–402.
99. Zissel G, Schlaak J, Schlaak M, Müller-Quernheim J. Regulation of cytokine release of alveolar macrophages by interleukin-4, interleukin-10, and transforming growth factor β. *Eur Cytokine Netw* 1996; **7:** 59–66.
100. Kehrl JH, Wakefield LM, Roberts AB, et al. Production of transforming growth factor β by human T lymphocytes and its potential role in the regulation of T cell growth. *J Exp Med* 1986; **163:** 1037–1050.
101. Lucas C, Bald LN, Fendly BM, *et al.* The autocrine production of transforming growth factor-$\beta_1$ during lymphocyte activation. *J Immunol* 1990; **145:** 1415–1422.
102. Yamamoto H, Hirayama M, Genyea C, Kaplan J. TGFβ mediates natural suppressor activity of IL-2-activated lymphocytes. *J Immunol* 1994; **152:** 3842–3847.
103. Koberda J, Grimm E, Moser R. Effect of anti-CD3/anti-CD28/interleukin-2 stimulation of mononuclear cells on transforming growth factor β inhibition of lymphokine-activated killer cell generation. *J Can Res Clin Oncol* 1993; **119:** 131–136.
104. Fan K, Ruan Q, Sensenbrenner L, Chen B. Transforming growth factor-beta 1 bifunctionally regulates murine macrophage proliferation. *Blood* 1992; **79:** 1679–1685.
105. Kulkarni AB, Huh CH, Becker D, *et al.* Transforming growth factor-β1 null mutation in mice causes excessive inflammatory response and early death. *Proc Natl Acad Sci USA* 1993; **90:** 770–774.
106. Shull MM, Ormsby I, Kier AB, *et al.* Targeted disruption of the mouse transforming growth factor-β1 gene results in multifocal inflammatory disease. *Nature* 1992; **359:** 693–699.
107. Wahl SM. Transforming growth factor β: the good, the bad, and the ugly. *J Exp Med* 1994; **180:** 1587–1590.
108. Wahl SM, Hunt DA, Wakefield LM, *et al.* Transforming growth factor (TGF-beta) induces monocyte chemotaxis and growth factor production. *Proc Natl Acad Sci USA* 1987; **84:** 5788–5792.
109. Brandes ME, Wakefield LM, Wahl SM. Modulation of monocyte type I transforming growth factor-beta receptors by inflammatory stimuli. *J Biol Chem* 1991; **266:** 19697–19703.
110. Denis M. Neutralization of transforming growth factor-beta 1 in a mouse model of immune-induced lung fibrosis. *Immunology* 1994; **82:** 584–590.
111. Santana A, Saxena B, Noble N, Gold L, Marshall B. Increased expression of transforming growth factor β isoforms ($\beta_1$, $\beta_2$, $\beta_3$) in bleomycin-induced pulmonary fibrosis. *Am J Respir Cell Mol Biol* 1995; **13:** 33–44.
112. Giri S, Hyde D, Hollinger M. Effect of antibody to transforming growth factor beta on bleomycin induced accumulation of lung collagen in mice. *Thorax* 1993; **48:** 959–966.
113. Castilla A, Prieto J, Fausto N. Transforming growth factor β1 and α in chronic liver disease. *N Engl J Med* 1991; **324:** 933–940.
114. Ansher M, Peters W, Reisenbichler H, Petros W, Jirtle R. Transforming growth factor β as a predictor of liver and lung fibrosis after autologous bone marrow transplantation for advanced breast cancer. *N Engl J Med* 1993; **328:** 1592–1598.
115. Rudnicka L, Varga J, Christiano AM, Iozzo RV, Jimenez SA, Uitto J. Elevated expression of type VII collagen in the skin of patients with systemic sclerosis: regulation by transforming growth factor beta. *J Clin Invest* 1994; **93:** 1709–1715.
116. Khalil N, O'Connor RN, Unruh HW, *et al.* Increased production and immunohistochemical localization of transforming growth factor-β in idiopathic pulmonary fibrosis. *Am J Respir Cell Mol Biol* 1991; **5:** 155–162.
117. Border WA, Noble NA. Transforming growth factor β in tissue fibroisis. *N Engl J Med* 1994; **331:** 1286–1292.
118. Bonner J, Badgett A, Lindroos P, Osornio-Vargas A. Transforming growth factor β1 downregulates the platelet-derived growth factor α-receptor subtype on human lung fibroblasts *in vitro.* *Am J Respir Cell Mol Biol* 1995; **13:** 496–505.
119. Bogdan C, Paik J, Vodovotz Y, Nathan C. Contrasting mechanisms for suppression of macrophage cytokine release by transforming growth factor-beta and interleukin-10. *J Biol Chem* 1992; **267:** 23301–23308.
120. Roth M, Golub S. Human pulmonary macrophages utilize prostaglandins and transforming growth factor β1 to suppress lymphocyte activation. *J Leukoc Biol* 1993; **53:** 366–371.
121. Kehrl JH, Roberts AB, Wakefield LM, Jakowlew S, Sporn MB, Fauci AS. Transforming growth factor β is an important immunomodulatory protein for human B lymphocytes. *J Immunol* 1986; **137:** 3855–3860.
122. Moses HL, Yang EY, Pietenpol JA. TGF-β stimulation and inhibition of cell proliferation: new mechanistic insights. *Cell* 1990; **63:** 245–247.
123. Ralph P, Nakoinz I, Sampson-Johannes A. Negative regulation of cytokines. Inhibition of the production of interleukin-1 and tumor necrosis factor. *Ann NY Acad Sci.* 1991; **628:** 326–336.
124. Baecher-Allan CM, Barth RK. PCR analysis of cytokine induction profiles associates with mouse strain variation in susceptibility to pulmonary fibrosis. *Reg Immunol* 1993; **5:** 207–217.
125. Torre-Amione G, Beauchamp RD, Koeppen H, *et al.* A highly immunogenic tumor transfected with a murine transforming growth factor type $\beta_1$ cDNA escapes immune surveillance. *Proc Natl Acad Sci USA* 1990; **87:** 1486–1490.
126. Prior C, Barbee RA, Evans PM, *et al.* Lavage versus serum measurement of lysozyme, angiotensin converting enzyme and other inflammatory markers in sarcoidosis. *Eur Respir J* 1990; **3:** 1146–1154.
127. Gilbert S, Steinbrech DS, Landas SK, Hunninghake GW. Amounts of angiotensin-converting enzyme mRNA reflect the burden of granulomas in granulomatous lung disease. *Am Rev Respir Dis* 1993; **148:** 483–486.
128. Selroos OB. Value of biochemical markers in serum for determination of disease activity in sarcoidosis. *Sarcoidosis* 1984; **1:** 45–49.
129. Specks U, Martin II WJ, Rohrbach MS. Bronchoalveolare lavage fluid angiotensin—converting enzyme in interstitial lung diseases. *Am Rev Respir Dis* 1990; **141:** 117–123.
130. Grönhagen-Riska C, Fyhrquist E, von Willebrandt E. Angiotensin I—converting enzyme: a marker of highly differentiated monocytic cells. *Ann NY Acad Science* 1986; **465:** 242–249.
131. Bazil V, Baudys M, Hilgert I, Stefanova ILMG, Zbrozek J, Horejsi V. Structural relationship between the soluble and membrane-bound forms of human monocyte surface glycoprotein CD14. *Mol Immunol* 1989; **26:** 657–662.
132. Goyert S, Ferrero E, Rettig W, Yenamandra A, Obata F, Le Beau M. The CD14 monocyte differentiation antigen maps to a region encoding growth factors and receptors. *Science* 1988; **239:** 497–500.
133. Lauener R, Goyert S, Geha R, Vercelli D. Interleukin 4 down-regulates the expression of CD14 in normal human monocytes. *Eur J Immunol* 1990; **20:** 2375–2381.
134. Tollerud D, Clark J, Brown L, *et al.* The influence of age, race, and gender on peripheral blood mononuclear-cell subsets in healthy nonsmokers. *J Clin Immunol* 1989; **9:** 214–222.
135. Pryjma J, Zembala M, Pituch-Noworolska A, Ernst M, van der Bosch J, Flad H. Monocyte-T-cell interactions in pokeweed mitogen-activated cultures. *Immunology* 1990; **71:** 397–403.
136. Robinson BWS, McLemore T, Crystal RG. γ interferon is spontaneously released by alveolar macrophages and lung T-lymphocytes in patients with pulmonary sarcoidosis. *J Clin Invest* 1985; **75:** 1488–1495.
137. Prior C, Haslam P. Increased levels of serum interferon-γ in pulmonary sarcoidosis and relationship with response to corticosteroid therapy. *Am Rev Respir Dis* 1991; **143:** 53–60.
138. Pforte A, Schiessler A, Gais P, *et al.* Expression of CD14 correlates with lung function impairment in pulmonary sarcoidosis. *Chest* 1994; **105:** 349–354.
139. Striz I, Zheng L, Wang J, Pokorná H, Bauer P, Costabel U. Soluble CD14 is increased in bronchoalveolar lavage of active sarcoidosis and correlates with alveolar macrophage membrane-bound CD14. *Am J Respir Crit Care Med* 1995; **151:** 544–547.
140. Homolka J, Lorenz J, Zuchhold HD, Müller-Quernheim J. Evaluation of soluble CD14 and serum neopterin as serum parameters of inflammatory activity of pulmonary sarcoidosis. *Clin Invest* 1992; **70:** 909–916.
141. Huber C, Batchelor JR, Fuchs D, *et al.* Immune response-associated production of neopterin: release from macrophages primarily under control of interferon-gamma. *J Exp Med* 1984; **160:** 310–316.
142. Eklund A, Blaschke E. Elevated serum neopterin in sarcoidosis. *Lung* 1986; **164:** 325–332.
143. Prior C, Frank A, Fuchs D, *et al.* Urinary neopterin excretion in pulmonary sarcoidosis: correlation to clinical course of disease. *Clin Chim Acta* 1988; **177:** 211–220.
144. Meliconi R, Lalli E, Borzi R, *et al.* Idiopathic pulmonary fibrosis: can cell mediated immunity markers predict clinical outcome. *Thorax* 1990; **45:** 536–540.
145. American Thoracic Society. Clinical role of bronchoalvoelar lavage in adults with pulmonary disease. *Am Rev Respir Dis* 1990; **142:** 481–486.
146. Costabel U, Bross KJ, Rühle KH, Lohr GW, Matthys H. Ia-like antigens on T-cells and their subpopulations in pulmonary sarcoidosis and hypersensitivity pneumonitis. *Am Rev Respir Dis* 1985; **131:** 337–342.
147. Costabel U, Bross KJ, Matthys H. Pulmonary sarcoidosis: assessment of disease activity by lung lymphocyte subpopulations. *Klin Wochenschr* 1983; **61:** 349–356.
148. Müller-Quernheim J, Krönke M, Strausz J, Schykowski M, Ferlinz R. Interleukin-2 receptor gene expression by bronchoalveolar lavage lymphocytes in pulmonary sarcoidosis. *Am Rev Respir Dis* 1989; **140:** 82–88.
149. duBois RM, Kirby M, Balbi B, Saltini C, Crystal RG. T-lymphocytes that accumulate in the lung in sarcoidosis have evidence of recent stimulation of the T-cell antigen receptor. *Am Rev Respir Dis* 1992; **145:** 1205–1211.
150. Saltini C, Hemler ME, Crystal RG. T lymphocytes compartmentalized on the epithelial surface of the lower respiratory tract express the very late activation antigen complex VLA-1. *Clin Immunol Immunopathol* 1988; **46:** 221–233.
151. Pinkston P, Bitterman PB, Crystal RG. Spontaneous release of interleukin-2 by lung T-lymphocytes in active pulmonary sarcoidosis. *N Engl J Med* 1983; **308:** 793–800.
152. Hunninghake GW, Bedell GN, Zavala DC, Monick M, Brady M. Role of interleukin-2 release by lung T-cells in active pulmonary sarcoidosis. *Am Rev Respir Dis* 1983; **128:** 634–636.
153. Müller-Quernheim J, Saltini C, Sondermeyer P, Crystal RG. Compartmentalized activation of the interleukin-2 gene by lung T-lymphocytes in active pulmonary sarcoidosis. *J Immunol* 1986; **137:** 3475–3483.
154. Curtis J, Kaltreider H. Characterization of bronchoalveolar lymphocytes during a specific antibody-forming cell response in the lungs of mice. *Am Rev Respir Dis* 1989; **139:** 393–400.
155. Semenzato GC, Trentin AR, Zambello R, *et al.* Evidence of cells bearing interleukin-2 receptor at the sites of disease activity in sarcoid patients. *Clin Exp Immunol* 1984; **57:** 331–337.
156. Hudspith BN, Flint KC, Geraint-James D, Brostoff J, McI Johnson N. Lack of immune deficiency in sarcoidosis: compartmentalisation of the immune response. *Thorax* 1987; **42:** 250–255.
157. Burmester G, Jahn B, Gramatzki M, Zacher J, Kalden J. Activated T cells *in vivo* and *in vitro*: divergence in expression of Tac and Ia antigens in the nonblastoid small T cells of inflammation and normal T cells activated *in vitro*. *J Immunol* 1984; **133:** 1230–1234.
158. Hofman FM, von Hanwehr RI, Dinarello CA, Mizel SB, Hinton D, Merrill JE. Immunoregulatory molecules and IL 2 receptors identified in multiple sclerosis brain. *J Immunol* 1986; **136:** 3239–3245.
159. Paine RI, Mody CH, Chawis A, Spahr MA, Turka LA, Toews GB. Alveolar epithelial cells block lymphocyte proliferation *in vitro* without inhibiting activation. *Am J Respir Cell Mol Biol* 1991; **5:** 221–229.
160. Pabst R, Binns R. Lymphocytes migrate from the bronchoalveolar space to regional bronchial lymph nodes. *Am Rev Respir Dis* 1995; **151:** 495–499.
161. Strauchen JA, Breakstone BA. IL-2 receptor expression in human lymphoid lesions. *Am J Pathol* 1987; **126:** 506–512.
162. Moller DR, Konishi K, Kirby M, Balbi B, Crystal RG. Bias toward use of a specific T-cell receptor β-chain variable region in a subgroup of individuals with sarcoidosis. *J Clin Invest* 1988; **82:** 1183–1191.
163. Grunewald J, Olerup O, Persson U, Öhrn BM, Wigzell H, Eklund A. T-cell receptor variable region gene usage by CD4+ and CD8+ T cells in bronchoalveolar lavage fluid and peripheral blood of sarcoidosis patients. *Proc Natl Acad Sci USA* 1994; **91:** 4965–4969.
164. Forman J, Klein J, Silver R, Liu M, Greenlee B, Moller D. Selective activation and accumulation of oligoclonal Vβ-specific T cells in active pulmonary sarcoidosis. *J Clin Invest* 1994; **94:** 1533–1542.
165. Tamura N, Moller D, Balbi B, Crystal R. Preferential usage of the T-cell antigen receptor beta-chain constant region C beta 1 element by lung T-lymphocytes of patients with pulmonary sarcoidosis. *Am Rev Respir Dis* 1991; **143:** 635–639.
166. Zissel G, Fleischer B, Müller-Quernheim J. Analyses of T cells from different compartments of patients with sarcoidosis. *Immunobiol* 1991; **183:** 244.
167. Bellocq A, Lecossier D, Pierre-Audigier C, Tazi A, Valeyre D, Hance AJ. T cell receptor repertoire of T lymphocytes recovered from the lung and blood of patients with sarcoidosis. *Am J Respir Crit Care Med* 1994; **149:** 646–654.
168. Dohi M, Yamamoto K, Masuko K, *et al.* Accumulation of multiple T cell clonotypes in lungs of healthy individuals and patients with pulmonary sarcoidosis. *J Immunol* 1994; **152:** 1983–1988.
169. Jones CW, Lake RA, Wijeyekoon JB, Mitchell DM, duBois RM, O'Hehir RO. Oligoclonal V gene usage by T lymphocytes in bronchoalveolar lavage fluid from sarcoidosis patients. *Am J Respir Cell Mol Biol* 1996; **14:** 470–477.
170. Burastero SE, Borgonovo B, Gaffi D, *et al.* The repertoire of T-lymphocytes recovered by bronchoaleolar lavage from healthy nonsmokers. *Eur Respir J* 1996; **9:** 319–327.
171. Pannetier C, Even J, Kourilsky P. T-cell repertoire diversity and clonal expansion in normal and clinical samples. *Immunol Today* 1995; **16:** 176–181.
172. Rao N, Naidu Y, Bell R, *et al.* Usage of T cell receptor β-chain variable gene is highly restricted at the site of inflammation in murine autoimmune uveitis. *J Immunol* 1993; **150:** 5716–5721.
173. Posnett DN, Schmelkin I, Burton DA, August A, McGrath H, Mayer LF. T cell antigen receptor V gene usage. Increases in Vβ8+ T-cells in Crohn's disease. *J Clin Invest* 1990; **85:** 1770–1776.
174. Jenkins R, Nikaein A, Zimmermann A, Meek A, Lipsky P. T cell receptor Vβ gene bias in rheumatoid arthritis. *J Clin Invest* 1993; **92:** 2688–2701.
175. Müller-Quernheim J, Pfeifer S, Kienast K, Zissel G. Spontaneous interleukin-2 release of bronchoalveolar lavage cells in sarcoidosis is a codeterminator of prognosis. *Lung* 1996; **174:** 243–254.
176. Grutters JC, Drent M, van Velzen-Blad H, van den Bosch JMM. Improvement of bronchial asthma during an episode of active sarcoidosis. A case report. *Am J Respir Crit Care Med* 1996; **153:** A282.
177. Jung T, Lack G, Schauer U, *et al.* Decreased frequency of interferon-γ- and interleukin-2-producing cells in patients with atopic diseases measured at the single cell level. *J Allergy Clin Immunol* 1995; **96:** 515–527.
178. Robinson DS, Hamid Q, Ying S, *et al.* Predominant TH2-like bronchoalveolar T-lymphocyte population in atopic asthma. *N Engl J Med* 1992; **326:** 298–304.
179. Holt PG, Kees UR, Shon-Hegrad MA, *et al.* Limiting-dilution analysis of T cells extracted from solid human lung tissue: comparison of precursor frequences for proliferative responses and lymphokine production between lung and blood T cells from individual donors. *Immunology* 1988; **64:** 649–654.
180. Saltini C, Winestock K, Kirby M, Pinkston P, Crystal RG. Maintenance of alveolitis in patients with chronic beryllium disease by beryllium-specific helper T cells. *N Engl J Med* 1989; **320:** 1103–1109.
181. Garlepp MJ, Rose AH, Dench JE, Robinson BW. Clonal analysis of lung and blood T cells in patients with sarcoidosis. *Thorax* 1994; **49:** 577–585.
182. Bäumer I, Zissel G, Schlaak M, Müller-Quernheim J. Th1/Th2 cell distribution in pulmonary sarcoidosis. (manuscript submitted).
183. Hoshino T, Itoh K, Gouhara R, *et al.* Spontaneous production of various cytokines except IL-4 from CD4+ T cells in the affected organs of sarcoidosis patients. *Clin Exp Immunol* 1995; **102:** 399–405.
184. Kelso A. Th1 and Th2 subsets: paradigms lost? *Immunol Today* 1995; **16:** 374–379.
185. Krug N, Madden J, A.E. R, *et al.* T-cell cytokine profile evaluated at the single cell level in BAL and blood in allergic asthma. *Am J Respir Cell Mol Biol* 1996; **14:** 319–326.
186. Mosmann TR, Cherwinski H, Bond MW, Giedlin MA, Coffman RL. Two types of murine helper T cell clone. I. Definition according to profiles of lymphokine activities and secreted proteins. *J Immunol* 1986; **136:** 2348–2357.
187. Mosmann TR, Coffman RL. $T_{H1}$ and $T_{H2}$ cells: different patterns of lymphokine secretion lead to different functional properties. *Ann Rev Immunol* 1989; **7:** 145–173.
188. Holtzmann MJ, Sampath D, Castro M, Look DC, Jayaraman S. The one-two of T helper cells: does interferon-γ knock out the Th2 hypothesis for asthma? *Am J Respir Cell Mol Biol* 1996; **14:** 316–318.
189. Racke MK, Scott DE, Quigley L, *et al.* Distinct roles for B7-1 (CD-80) and B7-2 (CD-81) in the inhibition of experimental allergic encephalomyelitis. *J Clin Invest* 1995; **96:** 2195–2203.
190. Kuchroo VK, Das MP, Brown JA, *et al.* B7-1 and B7-2 costimulatory molecules activate differentially the developmental pathways: application to autoimmune disease therapy. *Cell* 1995; **80:** 707–718.
191. Bucy RP, Panoskaltsis-Mortari A, Huang G-Q, *et al.* Heterogeneity of single cell cytokine gene expression in clonal T cell populations. *J Exp Med* 1994; **180:** 1251–1262.
192. Nemoz G, Frigent AF, Aloui R, *et al.* Impaired G-protein and cyclic nucleotide phosphodiesterase activity in T-lymphocytes from patients with sarcoidosis. *Eur J Clin Invest* 1993; **23:** 18–27.
193. Agostini C, Zambello R, Sancetta R, *et al.* Expression of tumor necrosis factor-receptor superfamily members by lung T lymphocytes in interstitial lung disease. *Am J Respir Crit Care Med* 1996; **153:** 1359–1367.
194. Kabelitz D, Pohl T, Pechhold K. Activation-induced cell death (apoptosis) of mature peripheral T lymphocytes. *Immunol Today* 1993; **14:** 338–339.
195. Alderson MR, Tough TW, Davis-Smith T, *et al.* Fas ligand mediates activation induced cell death in human T-lymphocytes. *J Exp Med* 1995; **181:** 71–77.
196. Grell M, Zimmermann G, Hülser D, Pfizenmaier K, Scheurich P. TNF receptors TR60 and TR80 can mediate apoptosis via induction of distinct signal pathways. *J Immunol* 1994; **153:** 1963–1972.
197. Rubin LA, Kurman CC, Fritz ME, *et al.* Soluble interleukin 2 receptors are released from activated human lymphoid cells. *J Immunol* 1985; **135:** 3172–3177.
198. Reske-Kunz AB, Osawa H, Josimovic-Alasevic O, Rüde E, Diamantstein T. Soluble interleukin-2 receptors are released by long term-cultured insulin-specific T cells transiently after contact with antigen. *J Immunol* 1987; **138:** 192–196.
199. Semenzato G, Cipriani A, Trentin L, *et al.* High serum levels of soluble interleukin-2 receptors in sarcoidosis. *Sarcoidosis* 1987; **4:** 25–27.
200. Lawrence EC, Brousseau KP, Berger MB, Kurman CC, Marcon L, Nelson DL. Elevated concentration of soluble interleukin-2 receptors in serum samples and bronchoalveolar fluids in active sarcoidosis. *Am Rev Respir Dis* 1988; **137:** 759–764.
201. Müller-Quernheim J, Pfeifer S, Strausz J, Ferlinz R. Correlation of clinical and immunologic parameters of the inflammatory activity of pulmonary sarcoidosis. *Am Rev Respir Dis* 1991; **144:** 1322–1329.
202. Ina Y, Takada K, Sato T, Yamamoto M, Noda M, Morishita M. Soluble interleukin 2 receptor in patients with sarcoidosis. Possible origin. *Chest* 1992; **102:** 128–133.
203. Herrmann F, Cannistra SA, Levine H, Griffin JD. Expression of interleukin 2 receptors and binding of interleukin 2 by γ interferon-induced human leukemic and normal monocytic cell. *J Exp Med* 1985; **162:** 1111–1116.
204. Pforte A, Brunner A, Gais P, *et al.* Concomitant modulation of serum-soluble interleukin-2 receptor and alveolar macrophage interleukin-2 receptor in sarcoidosis. *Am Rev Respir Dis* 1993; **147:** 717–722.
205. Strausz J, Müller-Quernheim J, Ferlinz R. Serumspiegel des sezernierten Interleukin-2 Rezeptor als ein Aktivittsparameter der Sarkoidose. *Dtsch med Wschr* 1989; **114:** 744–749.
206. Schmitt M, Wirtz H. Sarkoidose der Lunge: Klinischer Verlauf und entzündliche Aktivität. *Pneumologie* 1995; **49:** 226–232.
207. Peters U, Papadopoulos T, Müller-Hermelink H. MHC class II antigens on lung epithelia of human fetuses and neonates. Ontogeny and expression in lungs with histologic evidence of infection. *Lab Invest* 1990; **63:** 38–43.
208. Papadopoulos T, Sirtl K, Dämmrich J, Müller-Hermelink H. Expressionsmuster von Integrinen auf Alveolarepithelien in der fetalen und adulten Lunge und bei interstitiellen Lungenerkrankungen. *Verh Dtsch Ges Path* 1993; **77:** (in press).
209. Guzman J, Izumi T, Nagai S, Costabel U. ICAM-1 and integrin expression on isolated human alveolar type II pneumocytes. *Eur Respir J* 1994; **7:** 736–739.
210. Limper A, Colby V, Sanders M, Asakura S, Roche P, DeRemee R. Immunohistochemical localization of transforming growth factor-β1 in the nonnecrotizing granulomas of pulmonary sarcoidosis. *Am J Respir Crit Care Med* 1994; **149:** 197–204.
211. Kohno N, Awaya Y, Oyama T, *et al.* KL-6, a mucin-like glycoprotein, in bronchoalveolar lavage fluid from patients with interstitial lung disease. *Am Rev Respir Dis* 1993; **148:** 637–642.
212. Kallenberg C, Schilizzi B, Beaumont F, de Leij L, Poppema S, The T. Expression of class II major histocompatibility complex antigens on alveolar epithelium in interstial lung disease: relevance to pathogenesis of idiopathic pulmonary fibrosis. *J Clin Pathol* 1987; **40:** 725–733.
213. Papadopoulos T, Ionescu L, Dmmrich J, Toomes H, Müller-Hermelink H. Type I and type IV collagen promote adherence and spreading of human type II pneumocytes *in vitro*. *Lab Invest* 1990; **62:** 562–569.
214. Hawgood S. Surfactant: composition, structure and metabolism. In: Crystal R, West J, ed. *The Lung: Scientific Foundations*. New York: Raven Press, 1991: 247–261.
215. Williams MD, Wright JR, March KL, W.RII M. Human surfactant protein A enhances attachment of *Pneumocystis carinii* to rat alveolar macrophages. *Am J Respir Cell Mol Biol* 1996; **14:** 232–238.
216. Tenner A, Robinson SL, Borchelt J, Wright JR. Human pulmonary surfactant protein (SP-A), a protein structurally homologous to C1q, can enhance FcR- and CR1-mediated phagocytosis. *J Biol Chem* 1989; **164:** 13923–13928.
217. Tegtmeyer FK, Gortner L, Ludwig A, Brandt E. In vitro modulation of induced neutrophil activation by different surfactant preparations. *Eur Respir J* 1996; **9:** 752–757.
218. Ghio AJ, Fracica SL, Young SL, Piantodosi CA. Synthetic surfactant scavenges oxidants and protects against hyperoxic lung injury. *J Appl Physiol* 1993; **77:** 1217–1223.
219. Antal JM, Divis LT, Erzurum SC, Wiedemann HP, Thomassen MJ. Surfactant suppresses NF-κB activation in human monocytic cells. *Am J Respir Cell Mol Biol* 1996; **14:** 374–379.
220. Lesur O, Mancini M, Janot C, *et al.* Loss of lymphocyte modulatory control by surfactant lipid extracts from acute hypersensitivity pneumonitis: comparison with sarcoidosis and idiopathic pulmonary fibrosis. *Eur Respir J* 1994; **7:** 1944–1949.
221. Hamm H, Lührs J, Guzman y Rotaeche J, Costabel U, Fabel H, Bartsch W. Elevated surfactant protein A in bronchoalveolar lavage fluids from sarcoidosis and hypersensitivity pneumonitis patients. *Chest* 1994; **106:** 1766–1770.
222. Jobe AH. Pulmonary surfactant therapy. *N Engl J Med* 1993; **328:** 861–868.
223. Konishi K, Moller DR, Saltini C, Kirby M, Crystal RG. Spontaneous expression of the interleukin 2 receptor gene and presence of functional interleukin 2 on T-lymphocytes in the blood of individuals with active pulmonary sarcoidosis. *J Clin Invest* 1988; **82:** 775–781.
224. Harmsen AG, Muggenburg BA, Snipes MB, Bice DE. The role of macrophages in particle translocation form lungs to lymph nodes. *Science* 1985; **230:** 1277–1280.
225. Chilosi M, Menestrina F, Capelli P, *et al.* Immunohistochemical analysis of sarcoid granulomas. Evaluation of Ki 67+ and Interleukin-1+ cells. *Am J Pathol* 1988; **131:** 191–198.
226. Bice DE, Williams AJ, Muggenburg BA. Long-term antibody production in canine lung allografts: implications in pulmonary immunity and asthma. *Am J Respir Cell Mol Biol* 1996; **14:** 341–347.
227. Murayama J, Yoshizawa Y, Sato T, *et al.* A compartmentalized bias for T-cell receptor Vβ usage in summer-type hypersensitivity pneumonitis. *Int Arch Allergy Immunol* 1995; **107:** 581–586.
228. Pinkston P, Saltini C, Müller-Quernheim J, Crystal RG. Corticosteroid therapy suppresses spontaneous interleukin-2 release and spontaneous proliferation of lung T-lymphocytes of patients with active pulmonary sarcoidosis. *J Immunol* 1987; **139:** 755–760.
229. Terashima T, Matsubara H, Nakamura M, *et al.* Local Pseudomonas instillation induces contralateral lung injury and plasma cytokines. *Am J Respir Crit Care Med* 1996; **153:** 1600–1605.

ACKNOWLEDGEMENT. This work was supported by the German Federal Research Foundation, DFG (Mu 692/3-2 and Mu 692/5-1).

**Received 17 June 1996; accepted 19 June 1996**
